# A Late Pleistocene archaic human tooth from Gua Dagang (Trader’s Cave), Niah national park, Sarawak (Malaysia)

**DOI:** 10.1371/journal.pone.0338786

**Published:** 2025-12-10

**Authors:** Darren Curnoe, Mohammed S. Sauffi, Hsiao Mei Goh, Xue-feng Sun, Roshan Peiris

**Affiliations:** 1 School of Arts, Western Sydney University, Parramatta South, New South Wales, Australia; 2 School of Biological, Earth and Environmental Sciences, University of New South Wales, Sydney, New South Wales, Australia; 3 Sarawak Museum Department, Jalan P. Ramlee, Kuching, Sarawak, Malaysia; 4 Centre for Global Archaeological Research, Universiti Sains Malaysia, Penang, Malaysia; 5 School of Geography and Ocean Science, Nanjing University, Nanjing, China; 6 Department of Basic Sciences, Faculty of Dental Sciences, University of Peradeniya, Peradeniya, Sri Lanka; 7 College of Health Sciences, Vin University, Hanoi, Vietnam; Sapienza University of Rome: Universita degli Studi di Roma La Sapienza, ITALY

## Abstract

The rarity of Late Pleistocene hominin remains from Insular Southeast Asia (ISEA) has hampered our ability to understand a crucial episode of human evolutionary history, namely, the global dispersal of *Homo sapiens* from Africa. Moreover, recent discoveries indicate a surprising level of taxic diversity during this time with at least two species—*H. floresiensis* and *H. luzonensis*—endemic to the region when *H. sapiens* first arrived. A third hominin dubbed the ‘Denisovans’ is shown from DNA evidence to have interbred with the ancestors of contemporary Indigenous populations across ISEA, New Guinea and Australia. Yet, the Denisovans have not been identified from the fossil record of the area despite recent breakthroughs in this regard on mainland East Asia. New excavations by our team at the Trader’s Cave in the Niah National Park (‘Niah Caves’), northern Borneo, have yielded an isolated hominin upper central permanent incisor dated with Optically Stimulated Luminescence dating of sediments to about 52 − 55 thousand years ago. Specimen SMD-TC-AA210 has a massive crown absolutely and relative to its root size, the crown is wide (mesiodistally) and relatively short (labiolingually). Morphologically, it exhibits a very strong degree of labial convexity, pronounced shovelling, and the bulging basal eminence exhibits several upward finger-like projections. Labial enamel wrinking on the enamel-dentine junction is expressed as two large ridges exhibiting numerous spine-like projections, and the lingual extensions on the enamel surface of the basal eminence are expressed as six extensions. This combination of crown size and morphological traits is not normally found in *H. sapiens* and instead characterises archaic members of *Homo* such as *H. erectus*, *H. neanderthalensis* and Middle Pleistocene hominins sharing a clade with *H. heidelbergensis.* The Trader’s Cave tooth suggests that an archaic hominin population inhabited northern Borneo just prior to or coincident with the arrival of *H. sapiens* as documented at the nearby West Mouth of the Niah Great Cave.

## Introduction

### Palaeoanthropology of insular Southeast Asia

**Fig 1:**
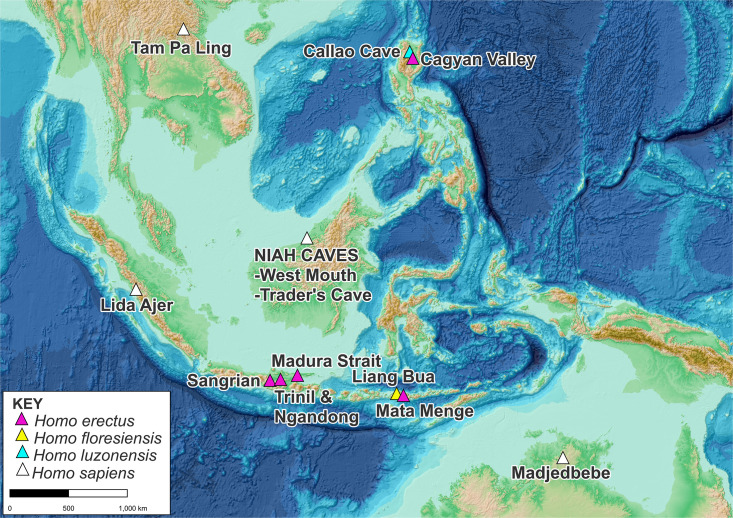
Map of southeast Asia indicating the location of the Trader’s Cave and the Pleistocene archaic human and early *H. sapiens* sites discussed in the text. Imagery reproduced from the GEBCO_2024 Grid, GEBCO Compilation Group (2022) GEBCO_2024 Grid (https://doi.org/10.5285/e0f0bb80-ab44-2739-e053-6c86abc0289c) which is in the public domain and may be used free of charge (https://www.gebco.net/data-products/gridded-bathymetry/terms-of-use).

Recent years have seen a fundamental shift in our understanding of Pleistocene human evolution in Insular Southeast Asia (ISEA). *Homo erectus* is now known to have inhabited the region after 1.5 million years ago (Ma) as documented at Sangiran in Indonesia [1] (Fig 1). By perhaps 1.48 Ma the species was widely dispersed across the area with archaeological evidence found at Calio on Sulawesi (Indonesia) [2], around 1.02 Ma at Wolo Sege on Flores [3] and in the Cagayan Valley on Luzon (Philippines) by about 709,000 years ago (ka) [4] (Fig 1). Evidence from Java further shows the species persisted through the Middle Pleistocene at Trinil (773−830 ka and <450 ± 110 ka) [5] with its final occurences during the early Late Pleistocene documented in submerged deposits in the Madura Strait (119 ± 27 ka to 162 ± 31 ka) [6] and at Ngandong (108–117 ka) [7] (Fig 1). Insular dwarf hominins which are likely to be phylogenetically related to *H. erectus* are now also known from Flores and Luzon in the form of *H. floresiensis* dated around 0.65–0.773 Ma at Mata Menge [8] and 60–100 ka at Liang Bua [9], and *H. luzonensis* at about 66.7 ± 1 ka from Callao Cave [10] (Fig 1).

The earliest fossil evidence for *H. sapiens* in ISEA has been dated 63–73 ka at Lida Ajer in Sumatra [11]. However, recent research suggests these findings may be complicated by uncertainties surrounding the stratigraphy of the site [12,13]. Offering support to an early arrival of *H. sapiens* in the region is the site of Tam La Ling (Laos) on the Southeast Asian mainland where human remains have been dated between 70 ± 3 ka and 77 ± 9 ka [14] (Fig 1). Further afield, the archaeological site of Madjebebe in northern Australia has provided Optically Stimulated Luminescence (OSL) sediment ages which imply human occupation back to 65 ka [15]. Again, questions have been raised about the reliability of this date due to possible stratigrapic complexities at the site [12,13].

All of these sites overlap in age with the deposits containing *H. floresiensis* and/or *H. luzonensis*, although, there is no evidence presently from the archaeological record that they interacted nor is there any indication of interbreeding from the genomes of contemporary populations [16]. While contemporary ISEA people do carry the genetic signtaures of interbreeding with archaic humans it most likely represents geneflow with *H. neanderthalensis*, as seen in all non-African populations, and the Denisovans, which reaches highest frequencies among Melanesians, Indigenous Australians and Insular Southeast Asians but is found also in ancient DNA samples from China and Mongolia [16–19]. Interestingly remains of the Denisovans are yet to be recognised in the human fossil record of ISEA although they have recently been identified on mainland East Asia [20,21].

Noteworthy also is the absence of evidence for *H. erectus* or any other archaic hominin in the archaeological record of Borneo: 1) in terms of its biogeography, Borneo is approximately six times larger than Java, located roughly mid-way between Java and Luzon, provided most of the land area of eastern Sunda during the glacial stages of the Middle and Late Pleistocene and was connected to mainland Southeast Asia during most of the later Pleistocene via corridors of Dipterocarp rainforest which served as species dispersal pathways [22,23]; 2) the longevity of the species in Southeast Asia (<1.5 million years) as well as growing knowledge about the species diversity, evolutionary ecology and biogeography of the *H. erectus* clade (e.g., [1–4,6–8]) implies more than sufficient time for multiple colonisation events from the southwest (Java/Sumatra) or north (mainland Southeast Asia) and possible speciation through isolation during glacial/interglacial cycles; and 3) the long history of archaeological research on the island back to the nineteenth century [24–27].

Systematic geological surveys aiming to locate Pleistocene exposures along the northern coastal region of Borneo were undertaken during the mid-twentieth century by the Sarawak Geological Survey, the Shell Company and Tom Harrisson but these were deemed unsuccessful [27]. Harrisson [27] briefly also describe a quartz hand axe recovered from a bauxite mine beside a mangrove near Kuching in Sarawak which he speculated might be associated with *Pithecanthropus* (i.e., *H. erectus*). But the original find seems to have been lost over the ensuing decades.

Presently, the earliest archaeological objects found in Borneo are the hand stencils at Lubang Jeriji Saléh in eastern Kalimatan (Indonesia), dated at least 51.8 ka [28], and in northern Borneo, stone artefacts from the Lobang Kuala (West Mouth) of the Niah Great Cave dated about 46–50 ka [29]. While several sites in the Mansuli Valley in southeast Sabah are suggested to contain Late Pleistocene sediments, their dating continues to be uncertain [30–33]. Regardless, the archaeological objects recovered at Lubang Jeriji Saléh, the West Mouth of the Niah Great Cave and the Mansuli Valley are all reasonably assumed to have been produced by *H. sapiens*.

The purpose of this contribution is to provide details of the stratigraphic context, dating, comparative morphology and significance of a newly discovered hominin tooth from the Gua Dagang (Trader’s Cave) in the World Heritage listed Niah National Park (‘Niah Caves’), Sarawak, Malaysia (Fig 1).

### Trader’s Cave history and speleology

**Fig 2:**
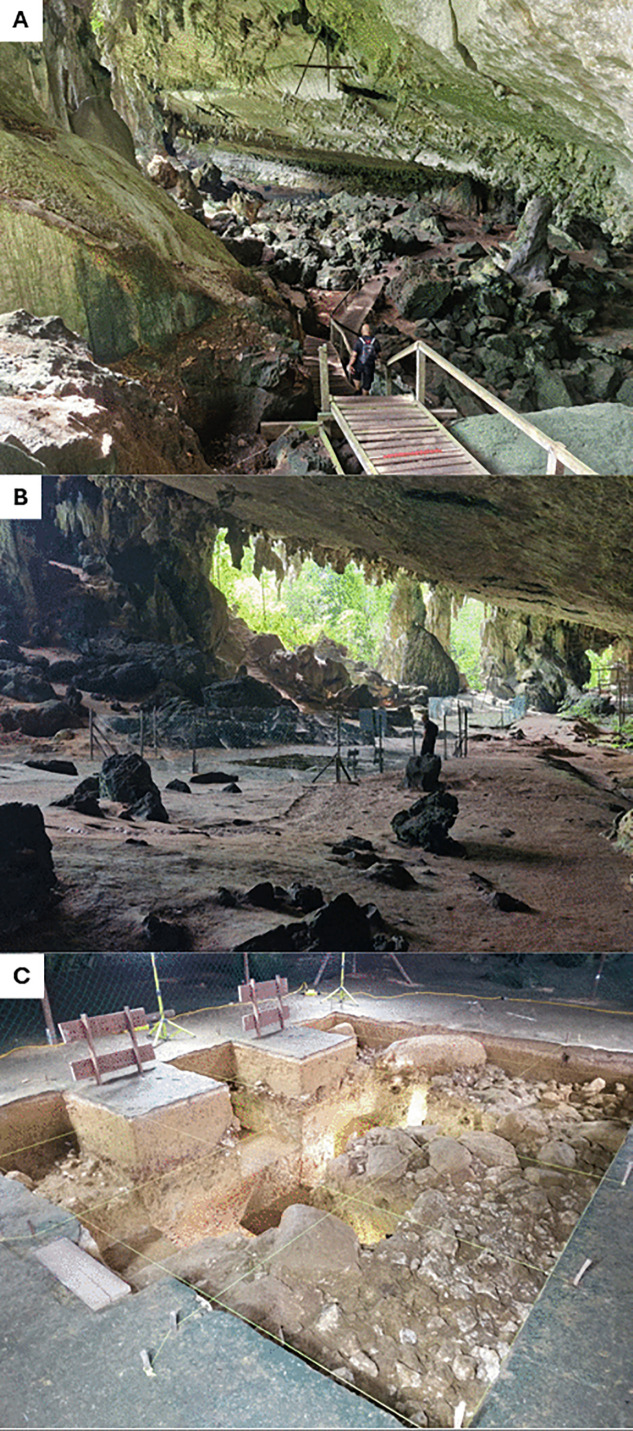
The Trader’s Cave. **A.** View north from the southern-most entrance to the cave; **B.** View northwest from central-east wall of the cave overlooking the two excavation areas taken during 2025 (Location A is in the foreground and Location B in the background); and **C.** Location A excavations at the end of the 2019 field campaign.

The cave receives its name from the bird (swiflet) nest trading camp in the cave which was abandoned in the 1980s and occupies the northern third of its main passage. The Trader’s Cave is a relic cave contained within the same steep-sided karst tower (Bukit Bekajang) as the Niah Great Cave (NGC) but stands alone from it located around 300 m north of the West Mouth of the NGC. It sits at an elevation of 37–57 m above current sea level [34]. The Trader’s Cave comprises a single large passage oriented in a roughly north-south direction with few dark zones (Fig 2). Its entire western side opens to a 100–200 m wide valley which separates the cave from the adjacent karst tower containing Mount Subis in the west. Within its western opening are 10 large speleothem columns or clusters of columns which lie within 12 m of the dripline [34]. A wide range of cave features (e.g., wall notches, ceiling slope and joints, and ceiling anastomoses) documenting successive phases of speleogenesis over a long period of time have been preserved due to the isolation of the Trader’s Cave from groundwater flow and other erosive forces [34] through tectonic uplift. The cave has previously been described as a remnant meander bend of a cave stream or a fluvially eroded undercut cliff [35,36]. Dodge-Wan [34] has proposed a model of development in which the Trader’s Cave was once a large domed cave perhaps over 90 m wide which subsequently decayed due to collapse during the Late Pleistocene followed by cliff retreat.

The horizontal length of the main passage is 150 m with an additional narrow rockshelter 50 m long in its northern entrance [34]. The passage is approximately 30 m wide throughout much of the cave but it reaches about 50 m in width at the southern end [34]. It extends to a height of approxiately 15 m in its southern part near the drip line on the west side of the cave. The lowest point of elevation of the Trader’s Cave is 13.6 m above the current dry season water level of the Sungai Subis (Subis River) and the highest point of its ceiling sits at 40 m above this level. The Subis River drains much of the Niah National Park, flowing into the South China sea about 17 km to the north. The southern section of the cave is wider and higher and contains a limestone bolder field caused by breakdown boulders [34] and accompanied by a shallow sediment deposit. The main passage has an estimated area of approxiately 5,856 m^2^ [34], is inclined south-north and west-east, and contains a sedimentary deposit of unknown thickness which contains the archaeological record outlined in the present study.

**Fig 3:**
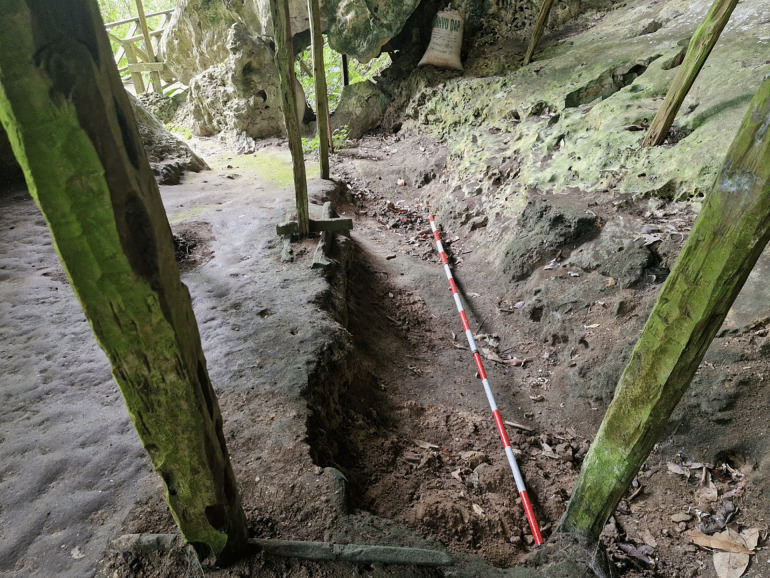
Harrisson test excavation trench in the Trader’s Cave (photographed in 2025, view looking north). The wooden poles are from the historic birds nest trader’s camp in the cave.

The Trader’s Cave was among the 37 caves “checked” by the Harrissons for potential archaeological deposits during the 1950s [27]. It was subject to testing in the form of a trench measuring about 400 cm x 100 cm x 30 cm in deposits in the northern (tourist) entrance of the cave (Fig 3). The excavations, undertaken in 1957, produced a sample of terrestrial mollusc shells from a shallow and archaeologically sterile deposit.

## Materials and methods

### Archaeological excavations

Over three field campaigns (Nov-Dec 2017, Apr 2018 and Feb-Apr 2019) of 15 weeks in total duration systematic excavations were undertaken at the Trader’s Cave. The excavation method was developed by DC and excavations were performed by hand using archaeological trowels by the authors (except X-FS) and several visiting archaeologists, Sarawak Museum staff and students under close supervision. Stratigraphic Layers 1–2 and Layer 4 were excavated as contexts while Layers 3 and 5–6 were excavated in 5 cm spits. Sediments were bucketed, weighed and wet sieved on site being passed through a 1 mm mesh (3 mm during the 2017 field campaign) using certified stainless steel test sieves from *NL Scientific* to ensure the effective capture of residuals. Full documentation of excavations and recording of finds was done using a purpose-designed digital recording system and photographic recording. Sediment samples were collected from each spit for archive and laboratory analysis. All *in situ* finds were recorded by logging their 3D (x,y,z) coordinates measured using steel measuring tapes and a Leica laser level system leveled with a fixed datum. All finds and sediment samples are stored in the Sarawak Museum Department in Kuching.

All necessary permits were obtained for the described study, which complied with all relevant regulations: State Planning Unit permit JKM.SPU/608-8/2/2 Vol.2, Sarawak Museum Department excavation permit A. 1007 and Sarawak Forestry Corporation Research Permit 001/2019 all of which were held by DC.

### Sediment analysis

Sediments were examined using a combination of section mapping, identification of sediment colour using the Munsell soil colour system and texture (ribbon) analysis to identify the sediment type.

### Geochronology

All Optically Stimulated Luminescence (OSL) dating samples were obtained in situ by hammering steel tubes into freshly cleaned vertical section walls. The tubes were immediately sealed with black plastic and tape to prevent light exposure and to ensure natural water was retained. All OSL measurements were conducted in the Luminescence Dating Laboratory at Nanjing University. Standard pre-treatment methods were used: the two outer ends of the samples were removed under subdued red light and used for water content and dose rate measurements. Then, the middle unexposed section was treated with 30% HCl and 30% H_2_O_2_ to remove carbonates and organic matter, respectively. After this, the coarser grain size fraction (63–90 μm) was extracted by wet sieving and the pure quartz grains (no significant IRSL signals) were obtained by normal acid etching (40% hydrogen fluoride for 40 min, follows 40 min 10% HCl rinse). Part of each sample was prepared by 40% fluosilicic acid for 3–7 days to obtain the pure 40–63 μm grains. The grains were then separated in density heavy liquid. Quartz grains were mounted as large (8 mm) aliquots on stainless steel discs. All luminescence measurements were made using Risø TL/OSL readers model DA-20 equipped with blue LEDs (470 nm, ~ 80 mW cm^2^) and infrared (IR) LEDs (870 nm, ~ 135 mW cm^2^). The OSL readers were also equipped with an accurately calibrated ^90^Sr/^90^Y beta source. Quartz OSL signals were collected through a 7.5 mm of Schott U-340 (UV) glass filter.

The radionuclide concentrations were measured by Gamma spectrometry with a high purity Ge-detector [37]. In situ water content (mass of moisture/dry mass) was determined by weighing the sample before and after drying and was assigned an absolute uncertainty of ± 7%. A small internal dose rate contribution from U and Th at 0.030 ± 0.015 and 0.06 ± 0.03 Gy/ka was also included. Using the revised dose rate conversion factors of Guerin et al. [38] and water content attenuation factors [39], the elemental concentrations were converted into an effective dose rate. Calculation of the cosmic dose rate is based on Prescott and Hutton [40].

**Fig 4:**
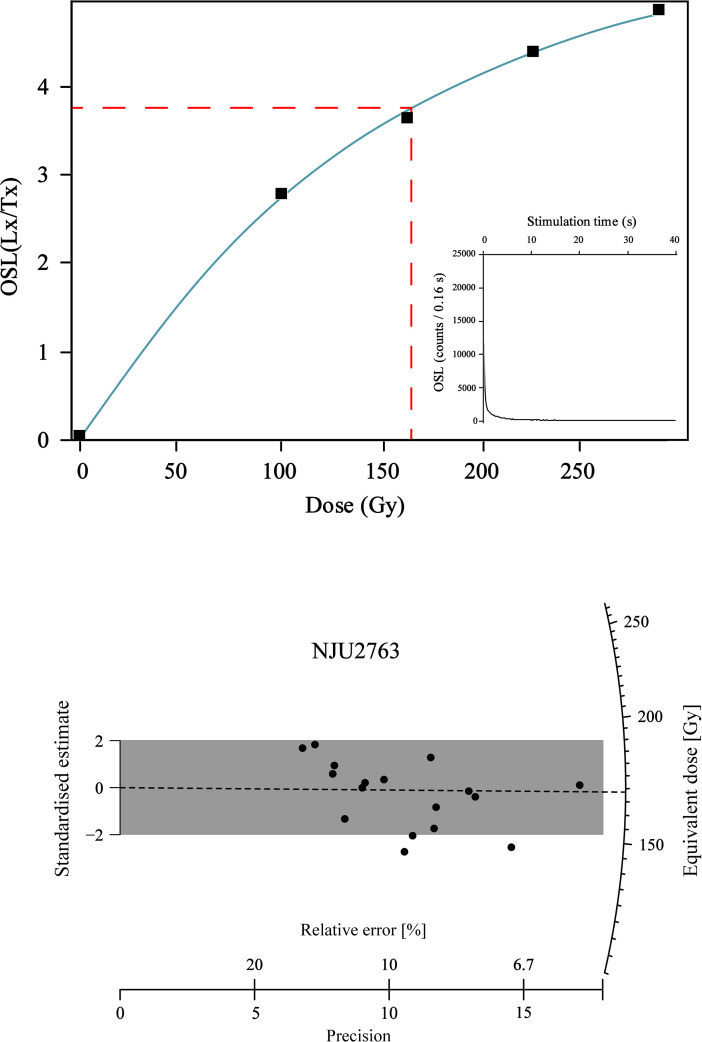
Representative OSL growth curve and decay curve (above) and De values of the OSL plotted on a radial plot (below) for sample NJU-2763.

Standard single aliquot regeneration dose (SAR-OSL) [41,42] methods were used to date the quartz. The aliquots were primarily preheated for 10 s at 260°C, while the response to the test dose was measured after a cut-heat to 220°C, followed by the optical stimulation 40 s at 125°C. All the dose response curves were fitted using saturating exponential functions in *Analyst* version 4.31.7 [43]. A representative OSL growth curve and decay curve and the De values of the OSL plotted on a radial plot for sample NJU-2763 are provided in Fig 4.

AMS radiocarbon (^14^C) dating was undertaken on in situ samples by Beta-Analytic Inc. Samples were calibrated by us using the IntCal20 Northern Hemisphere radiocarbon age calibration curve for charcoal [44] and the Marine20 radiocarbon age calibration curve [45] for the marine (oyster) shell samples recovered during excavations. Radiocarbon age calibration plots are provided in the supplementary information (S1 and S2 Figs). The following pre-treatments were applied to eliminate secondary carbon components because these components, if not eliminated, could result in a radiocarbon date that is too young or too old (https://www.radiocarbon.com/pretreatment-carbon-dating.htm). Charcoal pre-treatment: the sample was first gently crushed then dispersed in deionised water. It was then washed with hot HCl acid to eliminate carbonates followed by an alkali wash (NaOH) to remove secondary organic acids. The alkali wash was followed by a final acid rinse to neutralise the solution before drying. Chemical concentrations, temperatures, exposure times, and number of repetitions are dependent upon the sample. Each chemical solution was neutralised prior to application of the next solution. During these serial rinses, mechanical contaminants such as associated sediments and rootlets were eliminated. For shell samples, the following pre-treatment was applied: the calcareous material was first washed with deionised water, removing associated organic sediments and debris, where present. The material for radiocarbon dating was then crushed/dispersed and repeatedly subjected to HCl etches to eliminate the secondary carbonate components. In the case of thick shells, the surfaces were physically abraded prior to etching until the hard, primary core remained.

The ^230^Th dating work was performed at the Isotope Laboratory, Xi’an Jaiotong University using multi-collector inductively coupled plasma mass spectrometers (MC-ICP-MS) (Thermo-Finnigan Neptune-*plus*). Standard chemistry procedures were used to separate U and Th for dating [50]. A triple-spike (^229^Th–^233^U–^236^U) isotope dilution method was employed to correct for instrumental fractionation and determine U-Th isotopic ratios and concentrations. The instrumentation, standardisation and half-lives are reported in Cheng et al. [46,47]. All U-Th isotopes were measured on a MasCom multiplier behind the retarding potential quadrupole in the peak-jumping mode. The procedures described in Cheng et al. [46,47] were used to characterise the multiplier. Uncertainties in U-Th isotopic data were calculated offline at 2 σ level, including corrections for blanks, multiplier dark noise, abundance sensitivity, and contents of the same nuclides in spike solution. Corrected ^230^Th ages assume the initial ^230^Th/^232^Th atomic ratio of 4.4 ± 2.2x10^-6^, the values for a material at secular equilibrium with the bulk earth ^232^Th/^238^U value of 3.8.

### Hominin tooth studies

Details of comparative samples employed in the present study are provided in Table 1, and the dataset of raw values is provided in S3 Table. The specimens included possessed both mesiodistal (MD) and labiolingual (LL) dimensions of the crown and teeth which were evidently heavily worn were excluded. MD and LL and root heights for AA210 were measured by DC with a standard digital sliding caliper and recorded on multiple occasions to the nearest 0.1 mm following the methods of Tobias [48]. Interstitial wear is minimal and the standard deviation of the MD (0.06) and BL (0) was determined from three separate measurements by author DC. The square root of crown area (MD*LL) was used to represent overall crown size (rather than crown area) because it linearises area measurements which makes comparisons across diverse taxa straightforward while stabilising variance and bringing sample distributions closer to a normal distribution. Crown shape was described using the standard index LL/MD*100. Statistical testing was conducted using the PAST program [49]. The comparative sample sizes were mostly small with some samples shown to violate the assumption of normality, as indicated by Shapiro-Wilk tests. Thus, sample medians and interquartile ranges were used for comparative purposes. Kruskal-Wallis tests with post hoc (paired) Mann-Whitney tests were also undertaken on the hominin comparative samples to assess the taxonomic valency of the four variables (S4-S7 Tables). Due to its very small sample size (n3), *H. habilis* was excluded from these tests. The results were significant (p < 0.001) in all cases indicating the variables have disciminatory power among all of the samples included for comparison. To assess the possible outlier status of SMD-TC-AA210 with respect to the median of each sample, the modified z-score, which is a non-parametric verison of the z-test, was used employing the following formula:

**Table 1:** Comparative samples used for metric analysis (note: complete dataset available in S3 Table).

Taxon	Sample Name	Age	Sites Included	Source
*Homo sapiens*	Sri Lanka Recent	Recent	Modern Sri Lankans	[52]
	Medieval Hungary	Recent	Halimba	[53]
	Niah Caves Metal Age	Holocene	Lobang Tulang	Present study
	West Malaysian Late Prehistoric	Holocene	Gua Cha, Guar Kepah, Gua Kerbau, Gua Harimau	[54]
	European Mesolithic	Holocene	Aveline’s Hole, Henriksholm Bøgebakken, Hoëdic, Korsør Nor, Muge Arruda, Muge Moita, Ofnet, Sejrø, Skateholm, Téviec	[53]
	Eurasian Upper Palaeolithic	Late Pleistocene	Abri Pataud, Arene Candide, Barma Grande, Cap Blanc, Chuandong, Dolni Vestonice, Dushan, Grotte de Enfants, Huanglong Cave, La Ferrassie, Le Peyrat, Le Rois, Lida Ajer, Předmost, St. Germain La Rivière, Vindija, Tubo	[55–57,69]
	Middle Palaeolithic Humans	Late Pleistocene	Skhul, Qafzeh	[58–60]
*Homo neanderthalensis*	*Homo neanderthalensis*	Middle Pleistocene–Late Pleistocene	Amud, Carihuela Cave, Combe Grenal, Genay, Krapina, La Chaise de Vouthon – Abri Bourgeois Delaunay, La Ferassaie, La Quina, Marillac Neanderthal, Palomas, Piece C, Scladina, Shanidar, Tabun, Vergisson	[53]
*Homo heidelbergensis* s.l.	China Middle Pleistocene	Middle Pleistocene–Late Pleistocene	Dingcun, Jinnuishan, Panxian Dadong, Tongzi, Xujiayao	[56,61–63]
	Sima de Los Huesos	Middle Pleistocene–Late Pleistocene	Atapuerca Sima de los Husesos	[51]
*Homo floresiensis*	Mata Menge	Middle Pleistocene	Mata Menge	[3]
*Homo erectus* s.l.	*Homo erectus* s.l.	Lower Pleistocene–Middle Pleistocene	Dmanisi, Hexian, KNM-WT 15000, Longtan Cave, Meipu, Olduvai Gorge, Sangiran, Wushan, Yuanmou, Yunxian, Zhoukoudian	[53,61,64–68]
*Homo habilis*	*Homo habilis*	Lower Pleistocene	Olduvai Gorge	[53]
*Pongo pygmaeus*	*Pongo pygmaeus*	Late Pleistocene & recent	Central Sumatra, Hoa Binh	[69,70]


$$Modified\;z-score\;=\frac{\text{0}.\text{6}\text{7}\text{4}\text{5}(x_{i}\;-\;x\tilde{})}{\text{MAD}}$$

This method uses a constant to approximate the standard deviation (0.6745), the sample median ($x\tilde{}$) and the median absolute deviation (MAD) of each datapoint from the sample median. Modified z-scores >3.5 or <−3.5 were assumed to be statistically significant.

Crown morphology was scored using the Arizona State University Dental Anthropology System (ASUDAS [50]) combined with the modifications made by Martinón-Torres et al. [51] for the inclusion of Pleistocene human teeth.

Micro-CT imaging was performed at the Tyree X-ray facilities at the University of New South Wales using a Mark I HeliScan system (ThermoFisher). The Mark I HeliScan system contains a Hamamatsu tube with diamond window, a high-quality flatbed detector (3,072 × 3,072 pixels, 3.75 fps readout rate) and a helical scanning system. The scanning parameters were: X-Ray energy 80kV; X-ray current 91µA; exposure time 0.6; number of accumulations 5; filter 3Al on camera; and trajectory was helix. The 3D model was produced by importing the Micro-CT scan images into MIMICS (v. 12.02). This software allows the user to manually edit and segment 2D layers from Micro-CT data to create 3D geometry in the stereolithography (STL) format.

Tooth SMD-TC-AA210 is held by the Sarawak Musuem Department, Jalan P. Ramlee, Kuching, Sarawak, Malaysia.

## Results

### Sediments and dating

**Fig 5:**
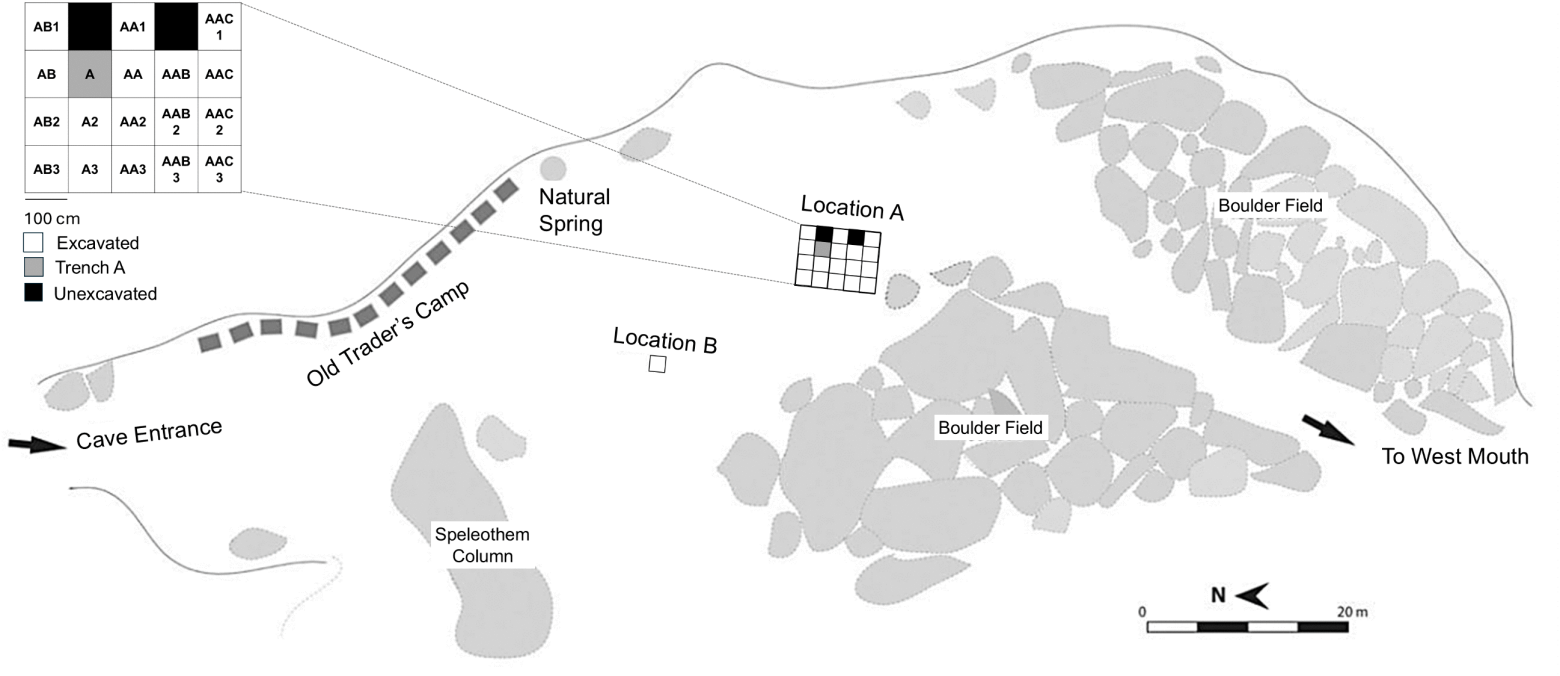
Plan of the Trader’s Cave highlighting the location of the excavation trenches for Location A and the single test pit for Location B.

Excavation Location A contains 20 trenches 18 of which have been excavated and to varying depths of 58–250 cm below surface as a result of the need to maintain safety, with no pit deeper than 150 cm below the adjacent pit surface to minimise the risks associated with sediment collapse (Fig 5). Location B contains a single test trench which was excavated to a depth of about 150 cm below surface (Fig 5). Only the findings from Location A will be reported here as the Location B data are yet to be analysed. The large amount of quartz contained within the Location A sediments allowed for the application of OSL dating (Table 2). AMS radiocarbon (^14^C) dating was applied to charcoal and marine shell samples collected in situ (Table 3). Uranium-series dating was applied to four samples collected from an in situ flowstone (Table 4).

**Table 2:** Results of Optically Stimulated Luminescence (OSL) dating.

Laboratory Number	Depth (cm)	H_2_O (wt%)	^40^K (ppm)*	^232^Th (ppm)*	^238^U (ppm)*	Total Dose Rate (Gy/ka)	Number of Aliquots	D_e_ (Gy)	OSL Age ± 1σ (ka)
Trench A									
NJU-2761	16.0	15.62	0.935	10.062	9.241	3.14 ± 0.26	12	103.1 ± 6.6	33 ± 3
NJU-2767	26.0	22.79	1.613	14.351	7.172	3.85 ± 0.32	9	136.3 ± 8.7	35 ± 4
NJU-2762	36.5	18.35	1.802	14.516	6.281	3.97 ± 0.33	12	210.1 ± 10.7	53 ± 5
NJU-2772	36.5	19.72	1.715	14.472	6.379	3.94 ± 0.32	11	205.9 ± 9.2	52 ± 5
NJU-2768	44.5	23.92	1.613	14.351	7.172	3.85 ± 0.32	11	203.2 ± 8.3	53 ± 5
NJU-2773	47.5	21.75	1.782	15.167	6.566	4.06 ± 0.33	11	221.1 ± 6.9	54 ± 5
NJU-2769	73.0	27.67	4.549	13.089	1.524	3.42 ± 0.28	12	218.2 ± 9.1	64 ± 6
NJU-2764	93.0	15.07	4.253	14.701	1.749	3.87 ± 0.32	12	233.5 ± 9.3	60 ± 6
NJU-2770	117.5	18.08	5.530	15.487	1.846	4.17 ± 0.34	11	256.4 ± 8.2	61 ± 6
NJU-2765	139.0	20.89	5.594	15.572	1.841	4.15 ± 0.34	12	262.8 ± 11.3	63 ± 6
NJU-2771	167.0	21.75	8.000	16.277	1.752	4.35 ± 0.36	12	324.1 ± 16.0	75 ± 7
NJU-2766	195.5	23.48	5.843	17.141	1.780	4.25 ± 0.35	11	318.9 ± 11.8	75 ± 7
NJU-3039	210.5	16.91	4.251	12.688	1.189	2.71 ± 0.19	8	215.24 ± 15.14	79 ± 8
NJU-3038	230.5	22.44	5.117	14.980	1.332	2.98 ± 0.19	2	341.34 ± 31.95	115 ± 13^†^
Trench AA									
NJU-2763	58.0	25.87	1.266	12.164	5.228	3.10 ± 0.25	17	170.4 ± 6.1	55 ± 5

*Radioelemental determinations conducted using inductively coupled plasma-mass spectrometry techniques.

† Saturated age.

**Table 3:** Results of AMS ^14^C dating.

Laboratory Code	Trench	Depth (cm)	Material	^14^C age ± 2σ(yr BP)	Calibrated agemedian	Calibrated age range (2σ, cal yr BP)	
						Min.	Max.
Beta-480575	A	12.5	Charcoal	33,530 ± 190	38,436	37,621	39,133
Beta-493583	AA	42.5	Shell	>43500	–	–	–
Beta-493581	AA	48	Shell	>43500	–	–	–
Beta-480577	A	52.5	Shell	>43500	–	–	–
Beta-493582	AA	52.5	Shell	34,690 ± 230	38,939	38,268	39,379
Beta-480578	A	57.5	Charcoal	20,070 ± 80	24,066	24,279	28,870
Beta-480580	A	67.5	Shell	>43500	–	–	–

**Table 4:** Results of uranium-series (U-Th) dating.

Sample code	^238^U (ppb)	± 2σ	^232^Th (ppb)	± 2σ	^230^Th/^232^Th (atomic x10^-6^)	± 2σ	^230^Th/^238^U (activity)	± 2σ	Age (uncorrected) ka	± 2σ	δ^234^U Initial (corrected)	± 2σ	Age (corrected) ka	± 2σ
AA2–222-A1	105.0	0.2	1474	30	2.2	1.8	0.3830	0.0021	52.8	0.4	−3	2	52.3	0.5
AAC-222-A2	39.9	0.1	1585	32	2.2	2.7	0.5242	0.0043	80.7	1.1	3	3	79.5	1.3
AAC-222-A3	71.2	0.1	1007	20	7.8	1.9	0.4114	0.0031	57.2	0.6	9	2	56.7	0.7
AAC-222-B1	109.0	0.2	6288	126	42.3	2.1	0.9006	0.0037	210.9	3.0	76	4	209.3	3.2
AAC-222-B2	117.7	0.2	8505	170	38.2	1.8	0.8859	0.0022	203.9	1.9	58	3	201.8	2.4
AAC-222-B3	117.1	0.2	3965	80	19.4	2.2	0.8696	0.0030	206.2	2.7	35	4	205.2	2.7

**Fig 6:**
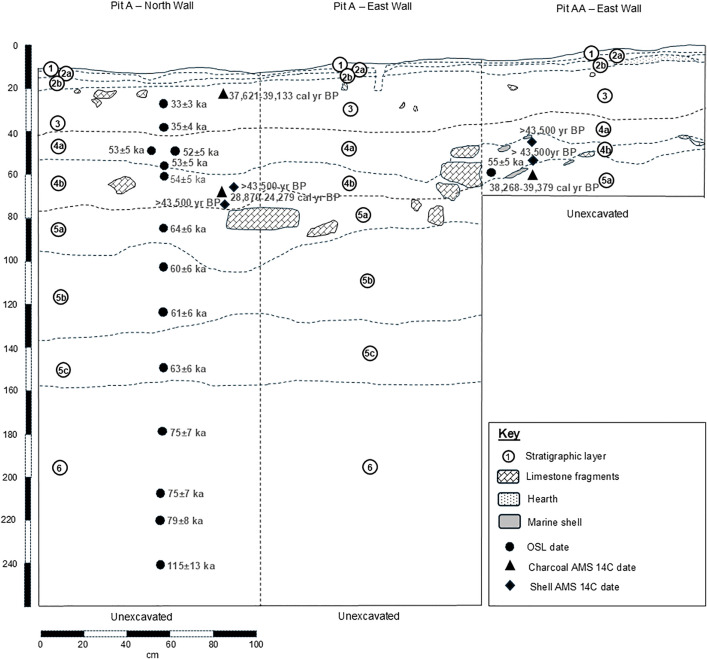
Section profiles for Trench A (North and East walls) and Trench AA (East wall) indicating OSL and AMS ^14^C dating results.

Ten sediment layers have been mapped within the section walls of the excavated trenches and they are summarised in Fig 6 and Table 5. The sediments are undisturbed except for the occasional ocurrence of bioturbation from wasp burrowing (typically up to a maximum of 30 cm deep), two shallow postholes from historic occupation of the cave and a shallow (approx. 20 cm deep) and narrow (approx. 30 cm) trench dug through the cave in the 1990s to provide electrical power for a former light installation in the West Mouth which affects five excavation pits. All layers except Layers 1 and 2 contained faunal remains and we provide a preliminary list of finds from the 2017 amd 2018 field campaigns in S8 Table.

**Table 5:** Description of sediment layers documented in east section wall of trench S2E3.

Layer	Depth Below Surface	Typical Thickness	Soil Type	Munsell Colour	Dating Samples (Lab Numbers)
1	0–3 cm	3 cm	Fungus encrusted mixed clay, guano and plant matter	Very dark grayish green (5GY3/2)	Undated
2a	3–6 cm	3 cm	Silty loam	Very pale brown (10YR7/3)	Undated
2b	6–12 cm	6 cm	Silty loam	Very pale brown (10YR7/3) or yellowish brown (10YR5/6)	Undated
3	12–32 cm	20 cm	Clay loam	Yellowish brown (10YR5/6) or dark yellowish brown (10YR4/6)	NJU-2761, NJU-2767, Beta-480575
4a	32–54 cm	22 cm	Clay loam with extensive mottling	Yellowish brown (10YR5/6) or light yellowish brown (10YR6/4), reddish brown (5YR4/4) mottling (c25%)	NJU-2762, NJU-2772, NJU-2768, Beta-493583
4b	54–64 cm	10 cm	Clay loam with extensive limestone inclusions of moderate–large size	Yellowish brown (10YR5/6) or light yellowish brown (10YR6/4)	NJU-2773, NJU-2763, Beta-493581, Beta-480577, Beta-493582, Beta-480578, Beta-480580
5a	64–85 cm	21 cm	Clay or clay loam with extensive mottling	Brownish yellow (10YR6/6) with light gray (5Y7/1 and 5Y7/2), pale yellow (5Y7/3) and red (2.5YR4/6) mottling (total <10%)	NJU-2769
5b	85–122 cm	37 cm	Heavy clay with mottling	Light yellowish brown (10YR6/4) or brownish yellow (10YR6/6), light gray (5Y7/2) mottling (c10%) and red (2.5YR4/6) mottling (c20%)	NJU-2764, NJU-2770
5c	125–145 cm	20 cm	Heavy clay with extensive mottling	Light yellowish brown (10YR6/4) or brownish yellow (10YR6/6), light gray (5Y7/2) mottling (c40%) and red (2.5YR4/6) mottling (c30%)	NJU-2765
6	145– > 250 cm	>130 cm	Heavy clay with extensive mottling	Yellowish red (5YR5/6), light gray (5Y7/2) and red (2.5YR4/6) mottling (total c20%)	NJU-2771, NJU-2766, NJU-3039, NJU-3038

These will be briefly described within the description of the stratigraphic units which follows. All layers contained cultural or likely cultural materials but these are yet to be subject to lithic analysis and will be published at a later time.

The upper 12 cm of the sediments comprises Layer 1 and Layers 2a and 2b (Fig 6). They range in thickness from about 3 cm to 6 cm with Layer 1 being relatively uniform in thickness but Layers 2a and 2b varying somewhat across the site (Table 5). Layer 1 is a very dark grayish green clay mixed with guano and plant material and has a thickness of about 3 cm (Table 5). The transition between Layer 1 and Layer 2a is sharp (i.e., within 2–5 cm). Layers 2a and 2b are visually distinct from each other in the section walls and the boundary between them is abrupt. Layer 2a is a very pale brown silty loam and Layer 2b is a very pale brown to yellowish brown silty loam. Layers 1 and 2 are currently undated. At the base of Layer 2b the transition to Layer 3 is sharp.

Layer 3 is a yellowish brown or dark yellowish brown clay loam typically about 20 cm thick (Fig 6, Table 5). OSL dating in Trench A indicates these sediments accumulated between 33 ± 3 ka (NJU-2761) and 35 ± 4 ka (NJU-2767) (Table 2). A fragment of charcoal from within the upper part of Layer 3 of Trench A has been dated with AMS ^14^C at 37,621–39,133 cal BP (at 2σ; Beta-480575; Table 3). Layer 3 provided 4.2% of the faunal specimens analysed to date with the majority (65.0%) being shells from fresh water species or land snails. Other fauna include marine oyster shell fragments (20.0%), crocodile (10.0%) and turtle (5.0%) (S8 Table). The interface between Layer 3 and Layer 4a is sharp.

Layer 4 comprises two subunits (Layers 4a and 4b) which have been identified from differences in sediment composition, Munsell colour and mottling. The transition between each of them is sharp. Layer 4a is a yellowish brown or dark yellowish brown clay loam with reddish brown mottling. It is typically about 22 cm in thickness but varies somewhat across the site (Fig 6, Table 5). The sediments in this layer form a veneer above Layer 4b which is a palaeofloor comprising rounded (water weathered) limestone rock fragments and small boulders. Sediments within the intersticies surrounding the boulders and immediately beneath them are a yellowish brown or light yellowish brown clay loam with extensive inclusions of small–large size. Layer 4b is typically about 10 cm in thickness but some of the limestone boulders are up to about 30 cm in thickness. Layer 4b may have formed during the hypothesised Late Pleistocene collapse event as part of the destruction of a former large domed cave [34]. Layer 4a and 4b combined have provided 82.4% of the faunal specimens from the 2017–2019 excavations and comprised many complete and some fragementary oyster shells (68.0% of the Layer 4 total), turtle (13.7%), shells from fresh water species or land snails (4.6%), unidentified bone (4.6%), crocodile (4.4%) and large mammal (3.9%), with bat, other reptile and fish contributing a very small amount to the total sample (each 0.3%) (S8 Table).

OSL samples taken in Layers 4a and 4b of Trench A are within error and imply these stratigraphic units accumulated between 52 ± 5 ka (NJU-2772; upper Layer 4a; refer also NJU-2762 & NJU-2768) and 54 ± 5 ka (NJU-2773; upper Layer 4b). AMS ^14^C dating of marine shell recovered from sediments immediately above the palaeofloor provided infinite ages (>43,500 BP: Trench A Beta-480577 and Trench AA Beta-493581 and Beta-493583; Table 3) consistent with OSL sediment dating with one exception: a well-preserved and largely complete shell recovered from immediately on top of the palaeofloor in trench AA above the provenience of the human tooth dated 38,268–39,379 cal BP (at 2σ; Beta-493582; Table 3). The young age of this shell compared with the sediment ages is probably best explained by contamination from non-autochthonous carbon, an issue encountered also with AMS ^14^C samples from the West Mouth [29], as the shell was too large to have been moved through bioturbation. A fragment of charcoal recovered from about the middle of Layer 4b also provided the anomalously young age of 24,279–28,870 cal BP (at 2σ; Beta-480578; Table 3). In this case, either reworking from Layer 2 or 3 due to bioturbation, contamination from non-autochthonous carbon or a combination of both offer an explanation for its apparent anomalous age. A further AMS ^14^C date on marine shell recovered from sediments in Trench A at the base of the palaeofloor also provided an infinite age (>43,500 BP: Beta-480580; Table 3).

A large in situ flowstone was uncovered during excavations in the southern part of Location A in Trench AAC and it lies stratigraphically immediately above Layer 4a. U-Th dating was undertaken on three samples each from the upper and lower sections of flowstone. The upper part provided ages of 52.3 ± 0.5 ka (sample code AAC-222-A1), 79.5 ± 1.3 ka (AAC-222-A2) and 56.7 ± 0.7 ka (AAC-222-A3) (Table 4) with a mean age of 62.8 ± 0.8 ka. Samples from the lower part were dated 209.3 ± 3.2 ka (AAC-222-B1), 201.8 ± 2.4 ka (AAC-222-B2) and 205.2 ± 2.7 ka (AAC-222-B3) (Table 4) with a mean age of 205.4 ± 2.8 ka. The age difference between them suggests the flowstone formed in two distinct phases during the later Middle Pleistocene and mid-Late Pleistocene. One explanation for this offset is that the flowstone originally formed in the ceiling, or was associated with a speleothem column in the western opening of the cave, and subsequently dislodged to become incorporated into the cave’s sedimentary sequence. The Late Pleistocene flowstone layer apparently precipitated soon after Layer 5 of the stratigraphic sequence formed. Sediments associated with Layer 4 subsequently accumulated beneath the flowstone slightly before and after the formation of the cave palaeofloor (Layer 4b) during the larger collapse event posited by Doge-Wan [34].

The interface between Layers 4b and 5a is sharp. Layer 5a comprises a brownish yellow clay or clay loam with light gray, pale yellow and red mottling (Table 5). It is typically around 21 cm thick but varies slightly in thickness across the site. A single OSL date establishes these sediments in Trench A to be 64 ± 6 ka (NJU-2769) (Table 2). The transition between Layers 5a/5b and 5b/5c is also sharp. Layer 5b is the second thickest layer in the Trader’s Cave sequence with a typical thickness of about 37 cm. It comprises a light yellowish brown or brownish yellow heavy clay with light gray and red mottling (Table 5). Two OSL dates from Trench A place the age of these sediments between 60 ± 6 ka (NJU-2764) and 61 ± 6 ka (NJU-2770) (Table 2, Fig 6). Layer 5c comprises a light yellowish brown or brownish yellow heavy clay with extensive light gray and red mottling (Table 5). A single OSL date from the lower part of this layer suggests these sediments are 63 ± 6 ka (NJU-2765) (Table 2, Fig 6). Overall, the OSL dates for Layer 5 are somewhat inverted with the oldest date seen in Layer 5a and the youngest in Layer 5b. Still, all of the OSL dates from Layer 5 are within error and as such imply that they sample a relatively short time period (around 4,000 years) and were deposited more or less continuously. Layer 5 has provided just 5.1% of the total faunal sample with small numbers of oyster shell (29.2% of Layer 5 total), crocodile and large mammal (16.7%), fish and other (non-crocodile) reptile (each 12.5%), unidentified bone (8.3%) and fresh water and land snail shell (4.2%) (S8 Table).

The interface between Layer 5c and Layer 6 is sharp. Layer 6 has currently been exposed in three trenches across Location A. It is the thickest unit and comprises a > 130 cm thick yellowish red heavy clay with extensive light gray and red mottling (Fig 6, Table 5). Layer 6 is the most basal layer exposed so far and the cave floor is yet to be reached. OSL ages place this layer in Trench A between 75 ± 7 ka (NJU-2771 & NJU-2766) and at least 79 ± 8 ka (NJU-3039) with a stratigraphically deeper (230.5 cm below surface) saturated OSL age of 115 ± 13 ka (NJU-3038) (Table 2). Layer 6 was also largely devoid of faunal remains but did contain small numbers of fresh water and land snail shell (66.7% of Layer 6 total%), turtle (12.8%), oyster shell, crocodile and unidentified bone (each 5.1%), and large mammal and bat (each 2.6%) (S8 Table).

### Human tooth

In February 2019 we recovered a human tooth (SMD-TC-AA210 or AA210) in situ from the northern part of excavation trench AA at a depth of 60 cm below surface. This places it stratigraphically within Layer 4b with a corresponding sediment (OSL) age of 54 ± 5 ka (Fig 6). An additional OSL sample was collected from the east wall of trench AA at a depth of 58 cm and horizontally about 50 cm east of the proveniece of AA210 and it provided a date of 55 ± 5 ka (NJU-2763; Fig 6). To take account of the possibility the tooth could potentially have moved vertically (downward) due to bioturbation from wasp burrowing, we place the age of AA210 conservatively within the range of c52–55 ka.

**Fig 7:**
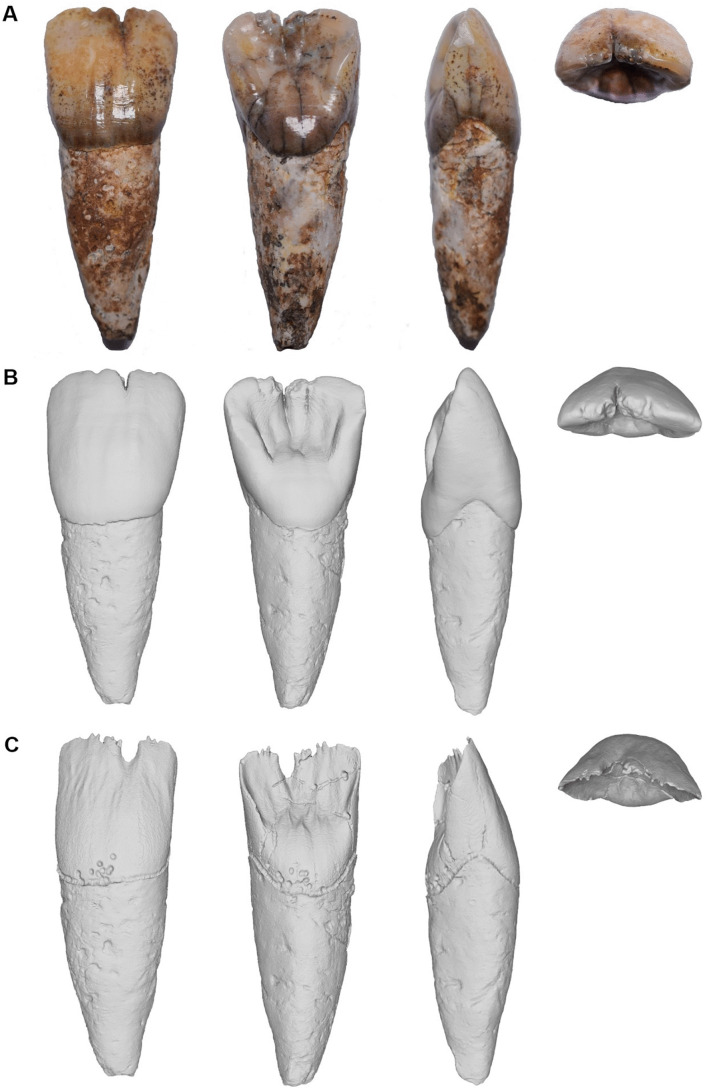
Specimen SMD-TC-AA210 (RI^1^) in labial (left), lingual (middle left), left lateral (middle right) and occulal (right) views. A: photographic image of the tooth. B: Rendered model of external surface from Micro-CT scans. C: Rendered model of enamel-dentine junction from Micro-CT scans.

**Fig 8:**
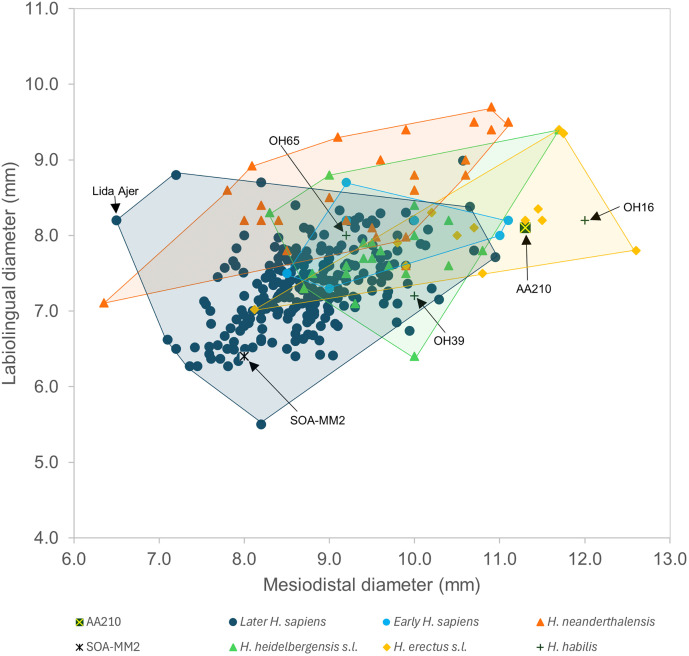
Mesiodistal and labiolingual diameters in SMD-TC-AA210 and comparative samples. Key: Late *H. sapiens* = Sri Lanka Recent, Hungry Medieval, Niah Caves Metal Age, European Mesolithic, West Malaysian Late Prehistroic and Eurasian Upper Palaeolithic; Early *H. sapiens* = Middle Palaeoltihic Humans. The remainder of the samples are defined in Table 1.

Specimen AA210 is a complete adult maxillary right central incisor (RI^1^) with slight wear on the occlusal surface but minimal interstitial wear (Molnar Stage 2 [71]), chipping medially along the occlusal surface and the possible presence of a labiogingival notch, a rare developmental defect in contemporary humans manifest as an enamel depression close to the cementoenamel junction [72] which appears to have gone unreported among Pleistocene hominins (Fig 7 and S9 Fig). The principal dimensions of its crown are minimally affected by wear and are provided along with the root dimensions in Table 6. Crown diameters, SQRT crown area and crown shape are compared in Tables 7 and 8. A bivariate plot comparing AA210 with a large sample of hominin incisors and defining the 2D-morphospace for *H. sapiens* and a range of other Pleistocene hominin species is provided in Fig 8.

**Table 6:** Metric dimensions of SMD-TC-AA210.

Variable	mm/%/mm^2^
Crown	
Mesiodistal (MD) (mm)	11.3
Labiolingual (LL) (mm)	8.1
Shape index (LL/MD) (%)	71.7
Crown area (mm^2^)	91.53
SQRT Crown area	9.6
Cervix	
Mesiodistal (MD) (mm)	8.2
Labiolingual (LL) (mm)	6.7
Root height	
Mesial (mm)	18.8
Distal (mm)	19.6
Labial (mm)	16.7
Lingual (mm)	16.1

**Table 7:** Comparison of the crown mesiodistal and labiolingual diameters of SMD-TC-AA210^†^.

	Mesiodistal Diameter (mm)						Labiolingual Diameter (mm)					
	n	Med.	IQR	MAD	Min.-Max.	Modified z-score	n	Med.	IQR	MAD	Min.-Max.	Modified z-score
SMD-TC-AA210		11.3						8.1				
*Homo sapiens*												
Sri Lanka Recent	121	8.6	0.74	0.37	7.4-9.8	**7.43**	121	7.2	0.56	0.28	6.3-8.2	3.25
Hungary Medieval	20	8.6	0.65	0.35	7.8-9.8	**7.71**	20	7.2	0.53	0.30	6.5-7.8	3.00
Niah Caves Metal Age	12	8.4*	0.43	0.20	7.2-9.1	**14.50**	12	7.7	1.00	0.55	6.4-8.8	0.73
West Malaysia Late Prehistoric	18	8.8	0.79	0.42	7.9-9.7	**6.02**	18	7.6	0.99	0.49	6.3-8.3	1.07
European Mesolithic	55	9.0	0.98	0.51	7.1-11.0	**4.51**	55	7.5*	0.56	0.28	6.3-9.0	2.32
Eurasian Upper Palaeolithic	42	9.0	0.85	0.50	6.5-10.7	**4.60**	42	7.6*	0.50	0.30	5.5-8.3	1.67
Middle Palaeolithic Humans	7	9.2	1.40	0.70	8.5-11.1	3.00	7	8.2	0.45	0.20	7.3-8.7	−0.50
*Homo neanderthalensis*	31	9.6	1.85	1.05	6.4-11.1	1.67	31	8.6	0.95	0.40	7.1-9.7	−1.25
*Homo heidelbergensis* s.l.												
China Middle Pleistocene	6	10.0	0.23	0.15	8.3-11.7	**8.67**	6	8.4	0.10	0.05	6.4-9.4	**−5.00**
Sima de los Husesos	20	9.5	0.55	0.30	8.7-10.8	**6.00**	20	7.7*	0.33	0.20	7.1-8.8	2.00
*Homo erectus* s.l.	16	10.8	1.35	0.73	8.1-12.6	0.76	16	8.2	0.44	0.23	7.0-9.4	−0.22
*Homo habilis*	3	10.0	1.40	0.80	9.2-12.0	1.63	3	8.0	0.50	0.20	7.2-8.2	0.50
*Pongo pygmaeus*	23	14.9	2.25	0.50	12.9-17.7	**−4.50**	23	12.3	1.75	0.90	10.9-14.4	**−4.67**

† Comparative sample compositions and data sources provided in Table 1. MAD = median absolute deviation. Significant modified z-scores given in bold.

* Sample is not normally distributed as indicated by a Shapiro-Wilk Test (p < 0.05).

**Table 8:** Comparison of the crown area and crown shape index of SMD-TC-AA210^†^.

	SQRT Crown Area (mm)						Crown Shape Index (%)					
	n	Med.	IQR	MAD	Min.-Max.	Modified z-score	n	Med.	IQR	MAD	Min.-Max.	Modified z-score
SMD-TC-AA210		9.6						71.7				
*Homo sapiens*												
Sri Lanka Recent	121	7.9*	0.48	0.24	6.8-8.8	**7.02**	121	85.0	5.2	3.5	70.9-97.4	**−3.75**
Hungary Medieval	20	8.0	0.52	0.32	7.1-8.4	**5.02**	20	84.5	6.8	3.9	72.4-96.2	−3.25
Niah Caves Metal Age	12	8.0	0.66	0.42	6.8-8.5	**3.81**	12	90.4*	7.9	4.3	74.4-122.2	**−4.39**
West Malaysia Late Prehistoric	18	8.0	0.93	0.35	7.1-8.9	**4.54**	18	86.6	9.1	4.9	74.4-99.0	−3.06
European Mesolithic	55	8.2	0.47	0.24	6.8-9.8	**5.85**	55	81.7*	8.2	4.5	67.8-93.9	−2.24
Eurasian Upper Palaeolithic	42	8.3	0.58	0.32	6.7-9.1	**4.04**	42	82.0*	7.5	3.9	67.1-126.2	−2.64
Middle Palaeolithic Humans	7	9.0	0.82	0.43	8.0-9.5	1.43	7	82.0	11.2	7.1	72.7-94.6	−1.45
*Homo neanderthalensis*	31	8.8	1.26	0.52	6.7-10.3	1.52	31	89.0*	13.0	4.8	76.8-112.0	**−3.64**
*Homo heidelbergensis* s.l.												
China Middle Pleistocene	6	9.2	0.71	0.47	8.0-10.5	0.85	6	82.3	3.6	1.8	64.0-100.0	**−5.80**
Sima de los Husesos	20	8.6	0.37	0.26	8.0-9.2	**3.59**	20	81.3*	4.0	2.2	72.2-97.8	**−4.44**
*Homo erectus* s.l.	16	9.3	0.69	0.43	7.6-10.5	0.59	16	76.5	7.6	3.9	61.9-86.5	−1.23
*Homo habilis*	3	8.6	0.72	0.09	8.5-9.9	**10.54**	3	72.0	9.3	3.7	68.3-87.0	−0.09
*Pongo pygmaeus*	23	13.5	1.96	0.80	12.1-16.0	**−4.86**	23	81.0	7.1	3.2	71.8-93.6	−2.85

† Comparative sample compositions and data sources provided in Table 1. MAD = median absolute deviation. Significant modified z-scores given in bold.

* Sample is not normally distributed as indicated by a Shapiro-Wilk Test (p < 0.05).

Overall, the Trader’s Cave tooth is distinguishable from *Pongo pygmaeus* by its smaller MD and LL diameters and SQRT crown area (Tables 7 and 8), labioloigually shorter crown (Table 7) and simpler crown morphology (see below). The AA210 values lie below its minimum for all four crown variables and the modified z-score implies that AA210 would be a outlier compared to the *P. pygmaeus* median for its MD (−4.50), LL (−4.67) and SQRT crown area (−4.86) (Tables 7 and 8). Thus, while its assignment to the Tribe Hominini appears to be clearcut the species assignment of AA210 is less straightforward.

The MD value for AA210 (11.3 mm) ranks only within the largest 3% of all values in our hominin sample, outside of (above) the range of values for all *H. sapiens* and within the largest 22% of archaic hominins (n374; Table 7 and S3 Table). Together, this highlights the large crown width of AA210. More specifically, compared to a wide range of hominins its MD dimension lies only within the range of China Middle Pleistocene (range 8.3–11.7 mm), *H. erectus* sensu lato (s.l.) (8.1–12.6 mm) and *H. habilis* (9.2–12.0 mm) (Table 7 and Fig 8). The MD value for AA210 is identical to the *H. erectus* specimen Sangiran S7-85. It is notably larger than the maximum for the Sri Lankan Recent and Hungrary Medieval (both 9.8 mm), Niah Caves Metal Age (9.1 mm), West Malaysian Late Prehistoric (Holocene) (9.7 mm), European Mesolithic (11.0 mm) and Eurasian Upper Palaeolithic (10.7 mm) samples (Table 7). Modified z-scores further imply that its MD diameter is an outlier compared to the median of the Sri Lankan Recent series (7.43), Hungary Medieval (7.71), Niah Caves Metal Age (14.50), West Malaysian Late Prehistoric (6.02), European Mesolithic (4.51) and Eurasain Upper Palaeolithic (4.60) samples (Table 7).

The LL value for AA210 (8.1 mm) ranks within the largest 19% of all values in our comparative sample, within the largest 9% of values for *H. sapiens* and within the largest 55% of archaic hominins. This indicates that the crown of AA210 is moderately long relative to other hominin I^1^s and long compared with most *H. sapiens*. More specifically, the crown of AA210 is most similar to the medians for Middle Palaeolithic Humans (Skhul and Qafzeh samples) and *H. erectus* s.l. (both 8.2 mm) (Table 7). The modified z-score is significant only for the China Middle Plesitocene (−5.00) implying AA210 is an outlier relative to the median of this sample (i.e., signficantly shorter).

The SQRT crown area for AA210 (9.6 mm) ranks within the largest 6% of all values in our hominin sample, within the largest 1% of values for *H. sapiens* and the largest 24% of archaic hominins highlighting its (relatively) large crown area. Specifically, its value lies within the range of *H. neanderthalensis* (6.7–10.3 mm), China Middle Pleistocene (8.0–10.5 mm), *H. erectus* s.l. (7.6–10.5 mm) and *H. habilis* (8.5–9.9 mm) (Table 8 and Fig 8). It is notably larger than the maximum for the Sri Lankan Recent series (8.8 mm), Hungary Medieval (8.4 mm), Niah Caves Metal Age (8.5 mm), West Malaysian Late Prehistoric (8.9 mm), European Mesolithic (9.8 mm) and Eurasain Upper Palaeolithic (9.1 mm) samples. Specimen AA210 would be an outlier (modified z > 3.50) compared to the median for all *H. sapiens* samples, except Middle Palaeolithic Humans, in addition to the Sima de los Huesos and *H. habilis* medians (Table 8).

The crown shape index of AA210 (71.7%) ranks within the smallest 3% of all values in our comparative sample, within the smallest 2% of *H. sapiens* and within the smallest 7% values of archaic hominins. Together, this highlights the unusually short crown of AA210 relative to its width. Its value does, however, lie within the range of the Sri Lanka Recent series (70.9–97.4%), European Mesolithic (67.8–93.8%), Eurasian Upper Palaeolithic (67.1–126.2%) and archaic hominins such as China Middle Pleistocene (64.0–100.0%), *H. erectus* s.l. (61.9–86.51%) and *H. habilis* (68.3–87.0%) (Table 8 and Fig 8). Modified z-scores indicate the AA210 crown shape index is an outlier compared to the median of the Sri Lankan Recent (−3.75), Niah Caves Metal Age (−4.39), *H. neanderthalensis* (−3.64), China Middle Pleistocene (−5.80) and Sima de los Huesos (−4.44) samples (Table 8).

The massive aspect of the Trader’s Cave tooth is apparent in lateral view, the crown being large compared with the size of the root (Fig 7 and S9 Fig). In the labial and lingual views, the crown of AA210 is trapezoidal in outline with curved sides becoming parallel as they approach the incisal edge. The mesial incisal angle is clearly straighter then the distal angle. In the occlusal aspect, the labial surface exhibits a very strong degree of convexity (modified ASUDAS Grade 5). Shovelling of the lingual surface is symmetric, but with a slightly thicker mesial marginal ridge, AA210 exhibiting moderate convexity (ASUDAS Grade 3). The degree of shovelling in the Trader’s Cave tooth exceeds that typically seen in *Australopithecus*, *H. habilis* and African *H. erectus* s.l. [48,64,73] and is closer to that characterising Eurasian Middle Pleistocene fossils and *H. neanderthalensis* [51,74]. Similarly, strong labial convexity, like that observed in AA210, is normally seen among Eurasian Middle Pleistocene hominins and is especially pronounced among European *H. heidelbergensis* s.l. and *H. neanderthalensis* [51,74]. While it is noteworthy that the range of variation in *H. sapiens* includes teeth with pronounced shovel shape its combination with very strong labial convexity distinguishes AA210 from the species [51,74].

The tuberculum dentale is bulging basal eminence (ASUDAS Grade 4) with three upward projections extending well onto the lingual surface and are especially clear in a rendered 3D model from Micro-CT scans (Fig 7). A rounded and elevated basal eminence is typical of Lower-Middle Pleistocene specimens from East Asia, European Middle Pleistocene groups and *H. neanderthalensis* [51,56,63,65,66,75]. Upward finger-like projections are characteristic of the upper central incisors of early African *Homo* and specimens from mainland East Asian *H. erectus* such as Yuanmou, Hexian and Zhoukoudian Loc. 1 but not Indonesian specimens from Sangiran [75]. Strikingly, the Trader’s Cave tooth more closely resembles Zhoukoudian incisors in this regard and differs from typical *H. sapiens* incisors.

The root is single, relatively short and thick, and subtriangular in transverse section. The surfaces (labial, lingual, mesial, and distal) are all reasonably well-defined. Root height to the base of the crown is provided for all surfaces in Table 6. In general, there is a faint longitudinal depression along the distal surface. From the labial and lingual surfaces, the apical third of the root diverges slightly distally and narrows considerably in its final apical quarter.

The enamel-dentine junction (EDJ) as revealed from a rendered model assembled from Micro-CT scans further highlights the complex morphology of AA210 strengthening its resemblances to archaic hominins (Fig 7). On the labial surface, palpable wrinkles on the outer enamel surface are expressed on the EDJ as two large ridges which narrow as they traverse superiorly. The upper surface of both exhibits numerous spine-like projections. On the lingual surface the expression of wrinkles and lingual ridges seen so clearly on the outer enamel surface are also evident. In this way, AA210 is remarkable in resembling the I^1^s from Zhoukoudian and Hexian [75]. There are three lingual extensions on the enamel surface and six on the EDJ which is more than seen on the Middle Pleistocene Panxian Dadong, Xujiayao and Tongzi I^1^s, similar to Zhoukoudian, but fewer than Hexian *H. erectus* teeth [56,63,65,66,75].

## Discussion

A maxillary central incisor (I^1^) recovered during excavations in the Trader’s Cave (specimen SMD-TC-AA210) exhibits a crown size and shape and external enamel surface and EDJ morphological traits that strongly resemble archaic humans. Specimen AA210 possesses a massive crown both absolutely and relative to its root size, and the crown is wide (MD) and relatively short (LL/MD). Morphologically, it exhibits a very strong degree of labial convexity, pronounced shovelling, and the bulging basal eminence exhibits several upward finger-like projections. The labial enamel wrinking is expressed on the EDJ as two large ridges which exhibit numerous spine-like projections, and the lingual extensions on the enamel surface of the basal eminence are expressed as six extensions on the EDJ. This combination of morphological traits is not usually found in *H. sapiens* and instead characterises archaic members of *Homo* such as *H. erectus*, *H. neanderthalensis* and the Middle Pleistocene hominins which occupy a clade with *H. heidelbergensis* [51,56,63,65,66,75].

It is problematic to assign an isolated tooth at the species level especially in the absence of apomorphic character traits. However, we can rule out membership of *H. neanderthalensis* on account of its known biogeographic distribution [76], and probably also *H. erectus* because the youngest examples have been found at Ngandong (Java) and date to more than 100 ka [6,7]. Besides, AA210 shows stronger morphological resemblances to mainland East Asian examples of this species such as from Zhoukoudian Loc. 1, Yuanmou, Hexian, Longtan Cave and Meipu. Yet, these specimens all belong to the Lower and Middle Pleistocene. The Trader’s Cave tooth also resembles Middle Pleistocene specimens from China, such as Xujiayao and Panxian Dadong [63], and some of these have recently been suggested to represent a mainland population of the Denisovans [20,21]. On this note, the Xiahe 2 specimen from Baishiya Cave establishes the existence of the Denisovans in Tibet as recently as 48–32 ka [77], While Pengu in Taiwan is of Late Pleistocene (10–70 ka) or Late-Middle Pleistocene (130–190) age [78]. Although *H. floresiensis* persisted on Flores until about 60 ka [9], the I^1^ crown of LB15/2 is too worn for morphological comparison. Still, its crown size is signficantly smaller than AA210 [79] as is the crown of the Middle Pleistocene specimen SOA-MM2 [3]. Similarly, *H. luzonensis* lacks preserved maxillary incisors, however, its teeth are overall also very small in size and most unlike AA210 in this regard [10,80].

The Trader’s Cave tooth suggests the existence of an unidentified archaic population at the Niah Caves in northern Borneo at around c52–55 ka. Remarkably, this is just prior to the earliest evidence for *H. sapiens* in the area, as documented in the nearby (~300 m away) site of the West Mouth of the Niah Great Cave where stone tools assumed to have been made by the species have been dated about 46–50 ka [29]. Further research should provide more evidence about the culture and economic activities of the archaic hominins that occupied the Trader’s Cave and will hopefully shed further light on their identity and the circumstances surrounding their disappearance.

## Supporting information

S1 Fig Calibrated AMS ^14^C charcoal ages. (DOCX)

S2 Fig Calibrated AMS ^14^C shell age. (DOCX)

S3 Table Raw data for comparative hominin tooth specimens. (DOCX)

S4 Table Results of Kruskal Wallis test and post hoc (pairwise) Mann-Whitney Bonferroni corrected p-values: mesiodistal diameter. (DOCX)

S5 Table Results of Kruskal Wallis test and post hoc (pairwise) Mann-Whitney Bonferroni corrected p-values: labiolingual diameter. (DOCX)

S6 Table Results of Kruskal Wallis test and post hoc (pairwise) Mann-Whitney Bonferroni corrected p-values: SQRT-crown area. (DOCX)

S7 Table Results of Kruskal Wallis test and post hoc (pairwise) Mann-Whitney Bonferroni corrected p-values: crown shape index. (DOCX)

S8 Table Preliminary results of faunal remains (individual finds) recovered from Trader’s Cave Location A excavations (2017–2019 campaigns). (DOCX)

S9 Fig SMD-TC-AA210 higlighting the location and extent of the labiogingival notch (white arrows). (DOCX)
