# Supplementary figures and images for: A Late Pleistocene archaic human tooth from Gua Dagang (Trader’s Cave), Niah national park, Sarawak (Malaysia)
Source: PLoS One. 2025 Dec 10;20(12):e0338786. doi: 10.1371/journal.pone.0338786 (PMC12694886; doi:10.1371/journal.pone.0338786)

**
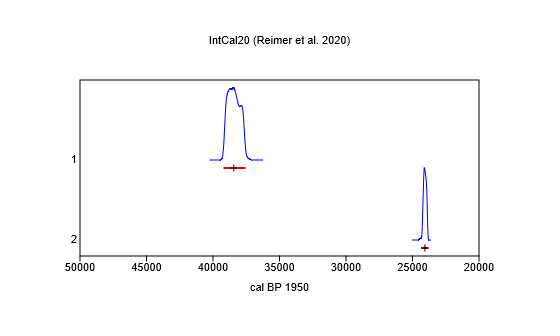
**

S1 Fig**. Calibrated AMS 14C charcoal ages.**

Supplement: S1 Fig — (DOCX) [file pone.0338786.s001.docx]

**
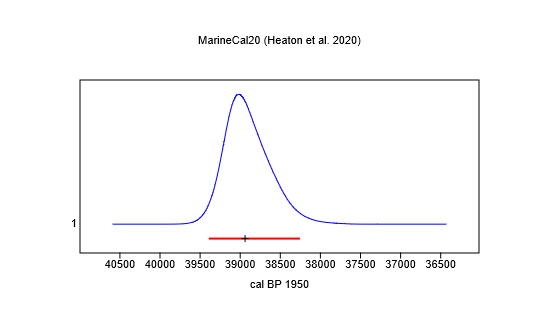
**

S2 Fig**. Calibrated AMS 14C shell age.**

Supplement: S2 Fig — (DOCX) [file pone.0338786.s002.docx]
