# Supplementary material for: A Late Pleistocene archaic human tooth from Gua Dagang (Trader’s Cave), Niah national park, Sarawak (Malaysia)
Source: PLoS One. 2025 Dec 10;20(12):e0338786. doi: 10.1371/journal.pone.0338786 (PMC12694886; doi:10.1371/journal.pone.0338786)
Supplement: S3 Table — (DOCX) [file pone.0338786.s003.docx]

**S3 Table. Raw data for comparative hominin tooth specimens.**

| **Specimen** | **MD (mm)** | **LL (mm)** | **SQRT-Area** | **Shape Index** |
| --- | --- | --- | --- | --- |
| Medieval Hungary |  |  |  |  |
| Halimba 1 | 9.8 | 7.1 | 8.3 | 72.4% |
| Halimba 45 | 7.8 | 6.5 | 7.1 | 83.3% |
| Halimba 115 | 8.4 | 7 | 7.7 | 83.3% |
| Halimba 119 | 8.2 | 7 | 7.6 | 85.4% |
| Halimba 143 | 9.1 | 7.2 | 8.1 | 79.1% |
| Halimba 264 | 8.8 | 7.2 | 8.0 | 81.8% |
| Halimba 338 | 8.6 | 6.6 | 7.5 | 76.7% |
| Halimba 352 | 8.9 | 7.8 | 8.3 | 87.6% |
| Halimba 393 | 8.4 | 7.6 | 8.0 | 90.5% |
| Halimba 401 | 8.6 | 6.7 | 7.6 | 77.9% |
| Halimba 438 | 8.1 | 7 | 7.5 | 86.4% |
| Halimba 440 | 7.9 | 7.6 | 7.7 | 96.2% |
| Halimba 449 | 9.4 | 7.5 | 8.4 | 79.8% |
| Halmiba 452 | 8.3 | 7.6 | 7.9 | 91.6% |
| Halimba 510 | 8.7 | 7.5 | 8.1 | 86.2% |
| Halimba 545 | 8.6 | 7.7 | 8.1 | 89.5% |
| Halimba 551 | 8.7 | 7.3 | 8.0 | 83.9% |
| Halimba 586 | 8 | 7.2 | 7.6 | 90.0% |
| Halimba 587 | 9 | 7.4 | 8.2 | 82.2% |
| Halimba 595 | 8 | 6.8 | 7.4 | 85.0% |
| Metal Age Niah Cave - Lobang Tulang |  |  |  |  |
| Unumbered | 8.8 | 8 | 8.4 | 90.9% |
| Unumbered | 8.7 | 8.1 | 8.4 | 93.1% |
| Unumbered | 7.2 | 6.5 | 6.8 | 90.3% |
| Unumbered | 8.6 | 6.4 | 7.4 | 74.4% |
| Unumbered | 8.2 | 8.7 | 8.4 | 106.1% |
| Unumbered | 8.6 | 8.4 | 8.5 | 97.7% |
| Unumbered | 8.4 | 7.6 | 8.0 | 90.5% |
| Unumbered | 8.4 | 7.2 | 7.8 | 85.7% |
| Unumbered | 7.2 | 8.8 | 8.0 | 122.2% |
| Unumbered | 9.1 | 7.8 | 8.4 | 85.7% |
| Unumbered | 8.3 | 7.5 | 7.9 | 90.4% |
| Unumbered | 8.2 | 7.1 | 7.6 | 86.6% |
| Mesolithic European |  |  |  |  |
| Aveline's Hole M1.11.163 | 9.1 | 6.8 | 7.9 | 74.7% |
| Henriksholm Bøgebakken 1 | 8.95 | 6.91 | 7.9 | 77.2% |
| Henriksholm Bøgebakken 19A | 8.7 | 7.7 | 8.2 | 88.5% |
| Henriksholm Bøgebakken 19C | 8.4 | 7.7 | 8.0 | 91.7% |
| Henriksholm Bøgebakken 5 | 8.75 | 7.69 | 8.2 | 87.9% |
| Henriksholm Bøgebakken 8 | 9 | 7.6 | 8.3 | 84.4% |
| Hoëdic 8 | 8.99 | 7.69 | 8.3 | 85.5% |
| Hoëdic 9 | 8.99 | 7 | 7.9 | 77.9% |
| Korsør Nor 1 | 9.1 | 7.3 | 8.2 | 80.2% |
| Muge Arruda 10.P | 8.61 | 7.66 | 8.1 | 89.0% |
| Muge Arruda 175.L | 9.17 | 7.07 | 8.1 | 77.1% |
| Muge Arruda 176a.L | 9.51 | 7.87 | 8.7 | 82.8% |
| Muge Arruda 177AA.L | 9.61 | 7.38 | 8.4 | 76.8% |
| Muge Arruda 3.L | 9.64 | 7.1 | 8.3 | 73.7% |
| Muge Arruda 902.L | 10.16 | 8.08 | 9.1 | 79.5% |
| Muge Arruda 910.L | 10.29 | 7.15 | 8.6 | 69.5% |
| Muge Arruda 911.L | 9.32 | 7.17 | 8.2 | 76.9% |
| Muge Arruda IVA.L | 8.89 | 7.26 | 8.0 | 81.7% |
| Muge Arruda XXV.E.L | 9.69 | 7.26 | 8.4 | 74.9% |
| Muge Moita 15.P | 9.68 | 7.74 | 8.7 | 80.0% |
| Muge Moita 17.P | 7.45 | 6.27 | 6.8 | 84.2% |
| Muge Moita 18.P | 7.1 | 6.62 | 6.9 | 93.2% |
| Muge Moita 19.L | 8.81 | 7.45 | 8.1 | 84.6% |
| Muge Moita 3.L | 9.67 | 7.47 | 8.5 | 77.2% |
| Muge Moita 5.L | 10.07 | 7.89 | 8.9 | 78.4% |
| Muge Moita 905.1001.L | 8.39 | 7.17 | 7.8 | 85.5% |
| Muge Moita XXXII.L | 9.6 | 7.58 | 8.5 | 79.0% |
| Muge Moita XXXIX.L | 9 | 7.16 | 8.0 | 79.6% |
| Ofnet 2476.3 | 9.92 | 7.36 | 8.5 | 74.2% |
| Ofnet 2481.8 | 8.62 | 7.12 | 7.8 | 82.6% |
| Ofnet 2484.11 | 9.69 | 7.47 | 8.5 | 77.1% |
| Ofnet 2486.13 | 8.9 | 6.8 | 7.8 | 76.4% |
| Ofnet 2487.14 | 8.69 | 6.63 | 7.6 | 76.3% |
| Ofnet 2493.21 | 9.55 | 7.76 | 8.6 | 81.3% |
| Ofnet 2501.29 | 8.6 | 7.6 | 8.1 | 88.4% |
| Sejrø 106956 | 8.93 | 7.4 | 8.1 | 82.9% |
| Skateholm 1 - 22 | 9.99 | 8.2 | 9.1 | 82.1% |
| Skateholm 1 - 25 | 8.23 | 7.11 | 7.6 | 86.4% |
| Skateholm 1 - 45 | 8.84 | 7.43 | 8.1 | 84.0% |
| Skateholm 1 - 47b | 9.1 | 7.48 | 8.3 | 82.2% |
| Skateholm 1 - 51 | 9.94 | 6.74 | 8.2 | 67.8% |
| Skateholm 1 - 53 | 9.79 | 6.85 | 8.2 | 70.0% |
| Skateholm 1 - 57 | 8.93 | 7.53 | 8.2 | 84.3% |
| Skateholm 1 - 59 | 8.51 | 7.67 | 8.1 | 90.1% |
| Skateholm 1 - 63a | 8.36 | 7.85 | 8.1 | 93.9% |
| Skateholm 2 - IV | 10.95 | 7.71 | 9.2 | 70.4% |
| Skateholm 2 - V | 10.57 | 8.99 | 9.7 | 85.1% |
| Skateholm 2 - VIII | 8.94 | 7.57 | 8.2 | 84.7% |
| Skateholm 2 - XA | 8.32 | 7.44 | 7.9 | 89.4% |
| Skateholm 2 - Xb | 10.13 | 7.87 | 8.9 | 77.7% |
| Skateholm 2 - XI | 9.5 | 7.27 | 8.3 | 76.5% |
| Skateholm 2 - XX | 10.65 | 8.38 | 9.4 | 78.7% |
| Téviec 14 | 8.21 | 6.76 | 7.4 | 82.3% |
| Téviec 3 | 8.7 | 7.6 | 8.1 | 87.4% |
| Téviec 4 | 8.9 | 7.8 | 8.3 | 87.6% |
| Late Palaeolthic Malaysian |  |  |  |  |
| Gua Cha B1 | 8.23 | 7.18 | 7.7 | 87.2% |
| Gua Cha B8 | 7.93 | 6.34 | 7.1 | 79.9% |
| Gua Cha H12 | 8.88 | 6.61 | 7.7 | 74.4% |
| Gua Cha H4 | 8.91 | 7.2 | 8.0 | 80.8% |
| Gua Harimau E-1 | 8.17 | 7.14 | 7.6 | 87.4% |
| Gua Kepah B324 | 9.12 | 8.33 | 8.7 | 91.3% |
| Gua Kepah C77 | 8.4 | 7.55 | 8.0 | 89.9% |
| Gua Kerbau 7 | 7.91 | 7.83 | 7.9 | 99.0% |
| Guag Kepah B320 | 8.62 | 6.77 | 7.6 | 78.5% |
| Guar Cha H10a | 8.46 | 7.35 | 7.9 | 86.9% |
| Guar Kepah B122 | 8.83 | 7.1 | 7.9 | 80.4% |
| Guar Kepah B175b | 9.54 | 8.16 | 8.8 | 85.5% |
| Guar Kepah B189 | 9.68 | 7.6 | 8.6 | 78.5% |
| Guar Kepah B260 | 8.77 | 8.08 | 8.4 | 92.1% |
| Guar Kepah B351 | 8.51 | 7.8 | 8.1 | 91.7% |
| Guar Kepah B353 | 9.6 | 8.29 | 8.9 | 86.4% |
| Guar Kepah C82c | 9.45 | 8.16 | 8.8 | 86.3% |
| Guar Kepah no num | 9.23 | 8.19 | 8.7 | 88.7% |
| Upper Palaeolithic *Homo sapiens* |  |  |  |  |
| Abri Pataud 2.2088 | 8.2 | 6.9 | 7.5 | 84.1% |
| Abri Pataud 2.2088 | 8.2 | 6.9 | 7.5 | 84.1% |
| Abri Pataud P1 (AP/89-2-62) | 8.6 | 7.8 | 8.2 | 90.7% |
| Arene Candide 1 | 9.5 | 8.2 | 8.8 | 86.3% |
| Arene Candide 3 | 9.3 | 7.6 | 8.4 | 81.7% |
| Arene Candide 8 | 9.7 | 7.6 | 8.6 | 78.4% |
| Barma Grande 3 | 9.3 | 7.6 | 8.4 | 81.7% |
| Cap Blanc 1 | 7.6 | 7 | 7.3 | 92.1% |
| Chuandong | 8.2 | 6.6 | 7.4 | 80.5% |
| Dolne Vestonice 13 | 9.3 | 8.2 | 8.7 | 88.2% |
| Dolne Vestonice 14 | 8.4 | 7.7 | 8.0 | 91.7% |
| Dolne Vestonice 15 | 9.3 | 8.1 | 8.7 | 87.1% |
| Dushan | 9.3 | 7.05 | 8.1 | 75.8% |
| Grotte des Enfants 6 | 9.8 | 8 | 8.9 | 81.6% |
| Huanglong Cave | 8.5 | 6.5 | 7.4 | 76.5% |
| La Fessassie LFb | 9.9 | 7.6 | 8.7 | 76.8% |
| Le Peyrat 5 | 5.7 | 6.6 | 6.1 | 115.8% |
| Le Rois 55.148f | 10.7 | 7.8 | 9.1 | 72.9% |
| Le Rois R50.5 | 9.6 | 7.7 | 8.6 | 80.2% |
| Le Rois R51.45 | 10.2 | 7.3 | 8.6 | 71.6% |
| Lida Ajer 1 | 6.5 | 8.2 | 7.3 | 126.2% |
| Předmost 1 | 9 | 7.8 | 8.4 | 86.7% |
| Předmost 10 | 9 | 7 | 7.9 | 77.8% |
| Předmost 14 | 9.8 | 7.8 | 8.7 | 79.6% |
| Předmost 3 | 8 | 8 | 8.0 | 100.0% |
| Předmost 4 | 8.8 | 7.3 | 8.0 | 83.0% |
| Předmost 5 | 9 | 7.4 | 8.2 | 82.2% |
| Předmost 9 | 9.2 | 7.4 | 8.3 | 80.4% |
| Predmosti 1 | 9 | 7.8 | 8.4 | 86.7% |
| Predmosti 10 | 9 | 7 | 7.9 | 77.8% |
| Predmosti 14 | 9.8 | 7.8 | 8.7 | 79.6% |
| Predmosti 2 | 9.6 | 7.5 | 8.5 | 78.1% |
| Predmosti 3 | 8.5 | 7.8 | 8.1 | 91.8% |
| Predmosti 4 | 8.8 | 7.3 | 8.0 | 83.0% |
| Predmosti 5 | 9 | 7.4 | 8.2 | 82.2% |
| Predmosti 9 | 9.2 | 7.4 | 8.3 | 80.4% |
| St. Germain La Rivière 12 | 9 | 7.7 | 8.3 | 85.6% |
| St. Germain La Rivière 15 | 9 | 7.6 | 8.3 | 84.4% |
| St. Germain La Rivière 16 | 9.5 | 7.4 | 8.4 | 77.9% |
| Tubo | 8.2 | 5.5 | 6.7 | 67.1% |
| Vindija 290 | 9.9 | 8.3 | 9.1 | 83.8% |
| Middle Palaeolithic *Homo sapiens* |  |  |  |  |
| Qafzeh 5 | 9 | 7.3 | 8.1 | 81.1% |
| Qafzeh 7 | 9.2 | 8.7 | 8.9 | 94.6% |
| Qafzeh 9 | 11.1 | 8.2 | 9.5 | 73.9% |
| Qafzeh Q20 | 11 | 8 | 9.4 | 72.7% |
| Skhul VII | 9.2 | 8.2 | 8.7 | 89.1% |
| Skhil IV | 10 | 8.2 | 9.1 | 82.0% |
| Skhul V | 8.5 | 7.5 | 8.0 | 88.2% |
| *Homo neanderthalensis* |  |  |  |  |
| Amud 1 | 9.2 | 8.2 | 8.7 | 89.1% |
| Carihuela Cave Pinar 7 | 10.6 | 9 | 9.8 | 84.9% |
| Cova Negra C.N. 7856 | 8.5 | 7.8 | 8.1 | 91.8% |
| Genay 1 | 8.4 | 8.2 | 8.3 | 97.6% |
| Krapina (?L) | 10.6 | 8.8 | 9.7 | 83.0% |
| Krapina (?L) | 10.7 | 9.5 | 10.1 | 88.8% |
| Krapina (?L) | 11.1 | 9.5 | 10.3 | 85.6% |
| Krapina 4 | 10 | 8.6 | 9.3 | 86.0% |
| Krapina 7 | 9.9 | 9.4 | 9.6 | 94.9% |
| Krapina 92 | 10.9 | 9.4 | 10.1 | 86.2% |
| Krapina D/D | 10 | 8.6 | 9.3 | 86.0% |
| Krapina F/H | 9.6 | 9 | 9.3 | 93.8% |
| Krapina MX E | 9.5 | 8.1 | 8.8 | 85.3% |
| Krapina MX I | 10.7 | 9.5 | 10.1 | 88.8% |
| Krapina MX K | 11.1 | 9.5 | 10.3 | 85.6% |
| Krapina MX Q | 10 | 8.8 | 9.4 | 88.0% |
| Krapina N/A | 10.9 | 9.7 | 10.3 | 89.0% |
| La Chaise de Vouthon - Abri Bourgeois Delaunay BD 10 | 8.2 | 8.4 | 8.3 | 102.4% |
| La Ferassaie | 9.9 | 7.6 | 8.7 | 76.8% |
| La Quina 5 | 8.09 | 8.92 | 8.5 | 110.3% |
| Marillac M13B | 9.55 | 7.98 | 8.7 | 83.6% |
| Neanderthal | 7.8 | 8.6 | 8.2 | 110.3% |
| Palomas 24 | 9.1 | 9.3 | 9.2 | 102.2% |
| Palomas 34 | 9.2 | 8 | 8.6 | 87.0% |
| Piece C | 9 | 8.5 | 8.7 | 94.4% |
| Scladina | 9.9 | 7.98 | 8.9 | 80.6% |
| Shanidar 2 | 8.2 | 8.2 | 8.2 | 100.0% |
| Tabun 1 | 8 | 8.2 | 8.1 | 102.5% |
| Tabun 3 | 9.2 | 8.2 | 8.7 | 89.1% |
| Vergisson 4-83 | 6.35 | 7.11 | 6.7 | 112.0% |
| Soa Basin |  |  |  |  |
| SOA MM2 | 8 | 6.4 | 7.2 | 80.0% |
| Middle Pleistocene China archaic |  |  |  |  |
| Tongzi | 10.3 | 8.3 | 9.2 | 80.6% |
| Dingcun | 8.3 | 8.3 | 8.3 | 100.0% |
| Jinniushan | 10 | 8.4 | 9.2 | 84.0% |
| Panxian Dadong | 10 | 6.4 | 8.0 | 64.0% |
| Xujiayao | 10 | 8.4 | 9.2 | 84.0% |
| Sima de los Huesos |  |  |  |  |
| AT-27 | 9.7 | 7.6 | 8.6 | 78.4% |
| AT-554 | 10.4 | 8.2 | 9.2 | 78.8% |
| AT-560 | 9.6 | 7.8 | 8.7 | 81.3% |
| AT-2752 | 8.8 | 7.5 | 8.1 | 85.2% |
| AT-1143 | 10.4 | 7.6 | 8.9 | 73.1% |
| AT-953 | 10.8 | 7.8 | 9.2 | 72.2% |
| AT-4320 | 9.3 | 7.1 | 8.1 | 76.3% |
| AT-165 | 9.2 | 7.5 | 8.3 | 81.5% |
| AT-199 | 9.6 | 7.8 | 8.7 | 81.3% |
| AT-8 | 10 | 8 | 8.9 | 80.0% |
| AT-54 | 9.9 | 7.5 | 8.6 | 75.8% |
| AT-146 | 9.5 | 7.7 | 8.6 | 81.1% |
| AT-197 | 9.2 | 7.5 | 8.3 | 81.5% |
| AT-278 | 9.4 | 7.7 | 8.5 | 81.9% |
| AT-280 | 9.4 | 7.9 | 8.6 | 84.0% |
| AT-1943 | 9.5 | 7.9 | 8.7 | 83.2% |
| AT-1958 | 9.2 | 7.6 | 8.4 | 82.6% |
| AT-3194 | 9.6 | 7.8 | 8.7 | 81.3% |
| AT-5615 | 8.7 | 7.3 | 8.0 | 83.9% |
| AT-3885 | 9 | 8.8 | 8.9 | 97.8% |
| *Homo erectus* |  |  |  |  |
| Dmanisi D2736 | 12.6 | 7.8 | 9.9 | 61.9% |
| Hexian | 11.7 | 9.4 | 10.5 | 80.3% |
| KNM-WT 15000 | 11.75 | 9.35 | 10.5 | 79.6% |
| Longtan Cave | 11.7 | 9.4 | 10.5 | 80.3% |
| Meipu | 10.2 | 8.3 | 9.2 | 81.4% |
| OH59 | 11.5 | 8.2 | 9.7 | 71.3% |
| Sangiran S7-1 | 10.5 | 8 | 9.2 | 76.2% |
| Sangiran S7-85 | 11.3 | 8.2 | 9.6 | 72.6% |
| Wushan | 8.12 | 7.02 | 7.5 | 86.5% |
| Yuanmou | 11.45 | 8.35 | 9.8 | 72.9% |
| Yunxian | 10.2 | 8.3 | 9.2 | 81.4% |
| Zhoukoudian No. 3 | 10.8 | 7.5 | 9.0 | 69.4% |
| Zhoukoudian No. 4 | 10.7 | 8.1 | 9.3 | 75.7% |
| Zhoukoudian No.1 | 9.9 | 7.6 | 8.7 | 76.8% |
| Zhoukoudian No.2 | 9.8 | 7.9 | 8.8 | 80.6% |
| Zhoukoudian PA66 | 10.7 | 8.1 | 9.3 | 75.7% |
| *Homo habilis* |  |  |  |  |
| OH16 | 12 | 8.2 | 9.9 | 68.3% |
| OH39 | 10 | 7.2 | 8.5 | 72.0% |
| OH65 | 9.2 | 8 | 8.6 | 87.0% |
| *Pongo pygmaeus* |  |  |  |  |
| Sibrambang Cave II471/1 | 14.9 | 12.8 | 13.8 | 85.9% |
| Sibrambang Cave II471/12 | 17.3 | 12.8 | 14.9 | 74.0% |
| Lida Ajer II1476/3 | 14.2 | 11.4 | 12.7 | 80.3% |
| Sibrambang Cave II471/14 | 16.3 | 12.3 | 14.2 | 75.5% |
| Site unknown II480/5 | 17.4 | 13.6 | 15.4 | 78.2% |
| Djamboe Cave II47/6 | 14.8 | 11.5 | 13.0 | 77.7% |
| Sibrambang Cave II471/17 | 15.7 | 13.3 | 14.5 | 84.7% |
| Sibrambang Cave II471/18 | 16.8 | 13.6 | 15.1 | 81.0% |
| Lida Ajer 9II1476/9 | 14.9 | 12 | 13.4 | 80.5% |
| Lida Ajer II1476/10 | 14.2 | 11.4 | 12.7 | 80.3% |
| Sibrambang Cave II471/111 | 14.1 | 13.2 | 13.6 | 93.6% |
| Lida Ajer II1476/12 | 15.6 | 14.3 | 14.9 | 91.7% |
| Sibrambang Cave II471/113 | 14.1 | 12.4 | 13.2 | 87.9% |
| Site unknown | 17.7 | 14.4 | 16.0 | 81.4% |
| Site unknown | 17 | 13.3 | 15.0 | 78.2% |
| Site unknown | 13.6 | 11.8 | 12.7 | 86.8% |
| Site unknown | 14.8 | 12 | 13.3 | 81.1% |
| Leiden Museum 2887.724 (24) | 12.9 | 11.6 | 12.2 | 89.9% |
| Zoological Musuem at Amsterdam A 9. Borneo (26) | 12.9 | 11.4 | 12.1 | 88.4% |
| Amsterdam Zoo 23-1-1922 (29) | 13.4 | 10.9 | 12.1 | 81.3% |
| Leiden Museum x. Borneo (30) | 15 | 12.1 | 13.5 | 80.7% |
| Leiden Museum z. western Sumatra (31) | 15.6 | 11.2 | 13.2 | 71.8% |
| Hoa Binh | 16.5 | 13.3 | 14.8 | 80.6% |
