# Supplementary material for: A Late Pleistocene archaic human tooth from Gua Dagang (Trader’s Cave), Niah national park, Sarawak (Malaysia)
Source: PLoS One. 2025 Dec 10;20(12):e0338786. doi: 10.1371/journal.pone.0338786 (PMC12694886; doi:10.1371/journal.pone.0338786)
Supplement: S4 Table — (DOCX) [file pone.0338786.s004.docx]

S4 Table**. Results of Kruskal Wallis test and post hoc (pairwise) Mann-Whitney Bonferroni corrected p-values: mesiodistal diameter.**

| H (chi^2^): | | | 116.1 | | |  |  |  |  |  |  |  |  |  |  |  |  |  |  |  |  |  |  |
| --- | --- | --- | --- | --- | --- | --- | --- | --- | --- | --- | --- | --- | --- | --- | --- | --- | --- | --- | --- | --- | --- | --- | --- |
| Hc (tie corrected): | | | 116.2 | | |  |  |  |  |  |  |  |  |  |  |  |  |  |  |  |  |  |  |
| p (same): | | | 3.045E-20 | | |  |  |  |  |  |  |  |  |  |  |  |  |  |  |  |  |  |  |
|  |  | | | |  | | |  | |  | |  | |  | |  | | |  | |  | |  |
|  | | SRL | | MED | | | NMA | | WMP | | MESO | | LPH | | MPH | | NEA | CMP | | SDH | | ERE | |
| SRL | |  | | 1 | | | 1 | | 1 | | 1.71E-06 | | 0.00013 | | 0.06371 | | 0.000549 | 0.08869 | | 3.76E-08 | | 2.33E-07 | |
| MED | | 1 | |  | | | 1 | | 1 | | 0.02997 | | 0.1688 | | 0.2595 | | 0.1471 | 0.2765 | | 0.000358 | | 0.000247 | |
| NMA | | 1 | | 1 | | |  | | 1 | | 0.01282 | | 0.08552 | | 0.1452 | | 0.2637 | 0.4632 | | 0.000443 | | 0.003903 | |
| WMP | | 1 | | 1 | | | 1 | |  | | 1 | | 1 | | 1 | | 1 | 0.4619 | | 0.03397 | | 0.000595 | |
| MESO | | 1.71E-06 | | 0.02997 | | | 0.01282 | | 1 | |  | | 1 | | 1 | | 1 | 1 | | 1 | | 6.73E-05 | |
| LPH | | 0.00013 | | 0.1688 | | | 0.08552 | | 1 | | 1 | |  | | 1 | | 1 | 0.4909 | | 0.4202 | | 4.36E-05 | |
| MPH | | 0.06371 | | 0.2595 | | | 0.1452 | | 1 | | 1 | | 1 | |  | | 1 | 1 | | 1 | | 1 | |
| NEA | | 0.000549 | | 0.1471 | | | 0.2637 | | 1 | | 1 | | 1 | | 1 | |  | 1 | | 1 | | 0.02501 | |
| CMP | | 0.08869 | | 0.2765 | | | 0.4632 | | 0.4619 | | 1 | | 0.4909 | | 1 | | 1 |  | | 1 | | 1 | |
| SDH | | 3.76E-08 | | 0.000358 | | | 0.000443 | | 0.03397 | | 1 | | 0.4202 | | 1 | | 1 | 1 | |  | | 0.006762 | |
| ERE | | 2.33E-07 | | 0.000247 | | | 0.003903 | | 0.000595 | | 6.73E-05 | | 4.36E-05 | | 1 | | 0.02501 | 1 | | 0.006762 | |  | |

Key: SRL = Sri Lankan Recent; MED=Medieval Hungary; NMA=Niah Caves Metal Age; WMP=West Malaysian Late Prehistoric; MESO=Mesolithic Europe; LPH=Late Palaeolithic Humans; MPH=Middle Palaeolithic Humans; NEA=*H. neadnerthalensis*; CMP=China Middle Pleistocene; SDH=Sima de Los Huesos; and ERE=*H. erectus* s.l.
