# Supplementary material for: A Late Pleistocene archaic human tooth from Gua Dagang (Trader’s Cave), Niah national park, Sarawak (Malaysia)
Source: PLoS One. 2025 Dec 10;20(12):e0338786. doi: 10.1371/journal.pone.0338786 (PMC12694886; doi:10.1371/journal.pone.0338786)
Supplement: S5 Table — (DOCX) [file pone.0338786.s005.docx]

S5 Table**. Results of Kruskal Wallis test and post hoc (pairwise) Mann-Whitney Bonferroni corrected p-values: labiolingual diameter.**

| H (chi2): | | | 129.1 | | |  |  |  |  |  |  |  |  |  |  |  |  |  |  |  |  |  |  |
| --- | --- | --- | --- | --- | --- | --- | --- | --- | --- | --- | --- | --- | --- | --- | --- | --- | --- | --- | --- | --- | --- | --- | --- |
| Hc (tie corrected): | | | 129.3 | | |  |  |  |  |  |  |  |  |  |  |  |  |  |  |  |  |  |  |
| p (same): | | | 6.938E-23 | | |  |  |  |  |  |  |  |  |  |  |  |  |  |  |  |  |  |  |
|  |  | | | |  | | |  | |  | |  | | |  | |  | |  | |  | | |
|  | | | SRL | | MED | | | NMA | | WMP | | MESO | | LPH | MPH | | NEA | | CMP | | SDH | | ERE |
| SRL | | |  | | 1 | | | 0.9427 | | 0.8279 | | 7.33E-03 | | 0.004183 | 0.01934 | | 4.96E-14 | | 0.2848 | | 4.76E-05 | | 2.54E-06 |
| MED | | | 1 | |  | | | 1 | | 1 | | 1 | | 0.5607 | 0.1247 | | 7.86E-07 | | 0.8772 | | 0.008674 | | 0.000738 |
| NMA | | | 0.9427 | | 1 | | |  | | 1 | | 1 | | 1 | 1 | | 0.07896 | | 1 | | 1 | | 1 |
| WMP | | | 0.8279 | | 1 | | | 1 | |  | | 1 | | 1 | 1 | | 0.000422 | | 1 | | 1 | | 0.5563 |
| MESO | | | 7.33E-03 | | 1 | | | 1 | | 1 | |  | | 1 | 0.5931 | | 8.28E-10 | | 0.7622 | | 0.4709 | | 1.21E-03 |
| LPH | | | 0.004183 | | 0.5607 | | | 1 | | 1 | | 1 | |  | 1 | | 4.72E-08 | | 0.4952 | | 1 | | 8.12E-03 |
| MPH | | | 0.01934 | | 0.1247 | | | 1 | | 1 | | 0.5931 | | 1 |  | | 1 | | 1 | | 1 | | 1 |
| NEA | | | 4.96E-14 | | 7.86E-07 | | | 0.07896 | | 0.000422 | | 8.28E-10 | | 4.72E-08 | 1 | |  | | 1 | | 0.000108 | | 1 |
| CMP | | | 0.2848 | | 0.8772 | | | 1 | | 1 | | 0.7622 | | 0.4952 | 1 | | 1 | |  | | 1 | | 1 |
| SDH | | | 4.76E-05 | | 0.008674 | | | 1 | | 1 | | 0.4709 | | 1 | 1 | | 0.000108 | | 1 | |  | | 0.2837 |
| ERE | | | 2.54E-06 | | 0.000738 | | | 1 | | 0.5563 | | 1.21E-03 | | 8.12E-03 | 1 | | 1 | | 1 | | 0.2837 | |  |

Key: SRL = Sri Lankan Recent; MED=Medieval Hungary; NMA=Niah Caves Metal Age; WMP=West Malaysian Late Prehistoric; MESO=Mesolithic Europe; LPH=Late Palaeolithic Humans; MPH=Middle Palaeolithic Humans; NEA=*H. neadnerthalensis*; CMP=China Middle Pleistocene; SDH=Sima de Los Huesos; and ERE=*H. erectus* s.l.
