# Supplementary material for: A Late Pleistocene archaic human tooth from Gua Dagang (Trader’s Cave), Niah national park, Sarawak (Malaysia)
Source: PLoS One. 2025 Dec 10;20(12):e0338786. doi: 10.1371/journal.pone.0338786 (PMC12694886; doi:10.1371/journal.pone.0338786)
Supplement: S6 Table — (DOCX) [file pone.0338786.s006.docx]

S6 Table**. Results of Kruskal Wallis test and post hoc (pairwise) Mann-Whitney Bonferroni corrected p-values: SQRT-crown area.**

| H (chi2): | | | 135.5 | | |  |  |  |  |  |  |  |  |  |  |  |  |  |  |  |  |  |  |
| --- | --- | --- | --- | --- | --- | --- | --- | --- | --- | --- | --- | --- | --- | --- | --- | --- | --- | --- | --- | --- | --- | --- | --- |
| Hc (tie corrected): | | | 135.6 | | |  |  |  |  |  |  |  |  |  |  |  |  |  |  |  |  |  |  |
| p (same): | | | 3.364E-24 | | |  |  |  |  |  |  |  |  |  |  |  |  |  |  |  |  |  |  |
|  |  | | | |  | | |  | |  | |  | | |  | |  | |  | |  | | |
|  | | | SRL | | MED | | | NMA | | WMP | | MESO | | LPH | MPH | | NEA | | CMP | | SDH | | ERE |
| SRL | | |  | | 1 | | | 1 | | 1 | | 1.71E-03 | | 6.44E-05 | 0.01457 | | 2.33E-11 | | 0.02453 | | 2.92E-08 | | 2.62E-07 |
| MED | | | 1 | |  | | | 1 | | 1 | | 0.9367 | | 0.1425 | 0.09713 | | 1.74E-05 | | 0.1418 | | 0.000137 | | 0.000268 |
| NMA | | | 1 | | 1 | | |  | | 1 | | 1 | | 1 | 0.5452 | | 0.01077 | | 0.9247 | | 0.03117 | | 0.00393 |
| WMP | | | 1 | | 1 | | | 1 | |  | | 1 | | 1 | 0.7178 | | 0.01685 | | 0.681 | | 0.7404 | | 0.002044 |
| MESO | | | 1.71E-03 | | 0.9367 | | | 1 | | 1 | |  | | 1 | 0.791 | | 9.93E-06 | | 0.6438 | | 0.009405 | | 1.78E-05 |
| LPH | | | 6.44E-05 | | 0.1425 | | | 1 | | 1 | | 1 | |  | 1 | | 1.05E-03 | | 1 | | 0.4616 | | 3.56E-05 |
| MPH | | | 0.01457 | | 0.09713 | | | 0.5452 | | 0.7178 | | 0.791 | | 1 |  | | 1 | | 1 | | 1 | | 1 |
| NEA | | | 2.33E-11 | | 1.74E-05 | | | 0.01077 | | 0.01685 | | 9.93E-06 | | 1.05E-03 | 1 | |  | | 1 | | 1 | | 1 |
| CMP | | | 0.02453 | | 0.1418 | | | 0.9247 | | 0.681 | | 0.6438 | | 1 | 1 | | 1 | |  | | 1 | | 1 |
| SDH | | | 2.92E-08 | | 0.000137 | | | 0.03117 | | 0.7404 | | 0.009405 | | 0.4616 | 1 | | 1 | | 1 | |  | | 0.004595 |
| ERE | | | 2.62E-07 | | 0.000268 | | | 0.00393 | | 0.002044 | | 1.78E-05 | | 3.56E-05 | 1 | | 1 | | 1 | | 0.004595 | |  |

Key: SRL = Sri Lankan Recent; MED=Medieval Hungary; NMA=Niah Caves Metal Age; WMP=West Malaysian Late Prehistoric; MESO=Mesolithic Europe; LPH=Late Palaeolithic Humans; MPH=Middle Palaeolithic Humans; NEA=*H. neadnerthalensis*; CMP=China Middle Pleistocene; SDH=Sima de Los Huesos; and ERE=*H. erectus* s.l.
