# Supplementary material for: A Late Pleistocene archaic human tooth from Gua Dagang (Trader’s Cave), Niah national park, Sarawak (Malaysia)
Source: PLoS One. 2025 Dec 10;20(12):e0338786. doi: 10.1371/journal.pone.0338786 (PMC12694886; doi:10.1371/journal.pone.0338786)
Supplement: S7 Table — (DOCX) [file pone.0338786.s007.docx]

S7 Table**. Results of Kruskal Wallis test and post hoc (pairwise) Mann-Whitney Bonferroni corrected p-values: crown shape index.**

| H (chi2): | | | 62.82 | | |  | | | |  |  |  |  |  |  |  |  |  |  |  |  |  |  |  |  |
| --- | --- | --- | --- | --- | --- | --- | --- | --- | --- | --- | --- | --- | --- | --- | --- | --- | --- | --- | --- | --- | --- | --- | --- | --- | --- |
| Hc (tie corrected): | | | 62.82 | | |  | | | |  |  |  |  |  |  |  |  |  |  |  |  |  |  |  |  |
| p (same): | | | 1.058E-09 | | |  | | | |  |  |  |  |  |  |  |  |  |  |  |  |  |  |  |  |
|  |  | | | |  | | |  | | |  | |  | | |  | |  | |  | |  | | |  |
|  | | | SRL | | MED | | | NMA | | WMP | | | MESO | | LPH | MPH | | NEA | | CMP | | SDH | | ERE |  |
| SRL | | |  | | 1 | | | 0.02808 | | 1 | | | 1.00E+00 | | 1.00E+00 | 1 | | 9.07E-05 | | 1 | | 6.41E-02 | | 1.38E-04 |  |
| MED | | | 1 | |  | | | 0.4999 | | 1 | | | 1 | | 1 | 1 | | 1.58E-01 | | 1 | | 1 | | 0.02687 |  |
| NMA | | | 0.02808 | | 0.4999 | | |  | | 1 | | | 1 | | 0.1025 | 1 | | 1 | | 1 | | 0.01733 | | 0.006989 |  |
| WMP | | | 1 | | 1 | | | 1 | |  | | | 1 | | 1 | 1 | | 1 | | 1 | | 1 | | 0.02223 |  |
| MESO | | | 1.00E+00 | | 1 | | | 1 | | 1 | | |  | | 1 | 1 | | 8.83E-02 | | 1 | | 1 | | 4.97E-02 |  |
| LPH | | | 1.00E+00 | | 1 | | | 0.1025 | | 1 | | | 1 | |  | 1 | | 3.05E-04 | | 1 | | 1 | | 5.54E-02 |  |
| MPH | | | 1 | | 1 | | | 1 | | 1 | | | 1 | | 1 |  | | 1 | | 1 | | 1 | | 1 |  |
| NEA | | | 9.07E-05 | | 1.58E-01 | | | 1 | | 1 | | | 8.83E-02 | | 3.05E-04 | 1 | |  | | 0.8257 | | 6.68E-05 | | 1.89E-05 |  |
| CMP | | | 1 | | 1 | | | 1 | | 1 | | | 1 | | 1 | 1 | | 0.8257 | |  | | 1 | | 1 |  |
| SDH | | | 6.41E-02 | | 1 | | | 0.01733 | | 1 | | | 1 | | 1 | 1 | | 6.68E-05 | | 1 | |  | | 0.6823 |  |
| ERE | | | 1.38E-04 | | 0.02687 | | | 0.006989 | | 0.02223 | | | 4.97E-02 | | 5.54E-02 | 1 | | 1.89E-05 | | 1 | | 0.6823 | |  |  |

Key: SRL = Sri Lankan Recent; MED=Medieval Hungary; NMA=Niah Caves Metal Age; WMP=West Malaysian Late Prehistoric; MESO=Mesolithic Europe; LPH=Late Palaeolithic Humans; MPH=Middle Palaeolithic Humans; NEA=*H. neadnerthalensis*; CMP=China Middle Pleistocene; SDH=Sima de Los Huesos; and ERE=*H. erectus* s.l.
