# Supplementary material for: A Late Pleistocene archaic human tooth from Gua Dagang (Trader’s Cave), Niah national park, Sarawak (Malaysia)
Source: PLoS One. 2025 Dec 10;20(12):e0338786. doi: 10.1371/journal.pone.0338786 (PMC12694886; doi:10.1371/journal.pone.0338786)
Supplement: S8 Table — (DOCX) [file pone.0338786.s008.docx]

**S8 Table. Preliminary results of faunal remains (individual finds) recovered from Trader’s Cave Location A excavations (2017-2019 campaigns).** Values in parenthesis are %-total for each faunal group in a given layer.

| Faunal group | Layer |  |  |  |  |  |  | |  |
| --- | --- | --- | --- | --- | --- | --- | --- | --- | --- |
|  | 1 | 2 | 3 | 4 | 5 | 6 | Total | %-Total | |
| Turtle | 0 | 0 | 1 (5.0%) | 53 (13.7%) | 0 | 5 (12.8%) | 59 | 12.5% | |
| Large mammal | 0 | 0 | 0 | 15 (3.9%) | 4 (16.7%) | 1 (2.6%) | 20 | 4.2% | |
| Bat | 0 | 0 | 0 | 1 (0.3%) | 0 | 1 (2.6%) | 2 | 0.4% | |
| Crocodile | 0 | 0 | 2 (10.0%) | 17 (4.4%) | 4 (16.7%) | 2 (5.1%) | 25 | 5.3% | |
| Other reptile | 0 | 0 | 0 | 1 (0.3%) | 3 (12.5%) | 0 | 4 | 0.8% | |
| Fish | 0 | 0 | 0 | 1 (0.3%) | 3 (12.5%) | 0 | 4 | 0.8% | |
| Unidentified | 0 | 0 | 0 | 18 (4.6%) | 2 (8.3%) | 2 (5.1%) | 22 | 4.7% | |
| Shell (oyster) | 0 | 0 | 4 (20.0%) | 264 (68.0%) | 7 (29.2%) | 2 (5.1%) | 277 | 58.8% | |
| Shell (fresh water/land snail) | 0 | 0 | 13 (65.0%) | 18 (4.6%) | 1 (4.2%) | 26 (66.7%) | 58 | 12.3% | |
| Total count | 0 | 0 | 20 | 388 | 24 | 39 | 471 | - | |
| %-Total per Layer | 0 | 0 | 4.2% | 82.4% | 5.1% | 8.3% | - | - | |
