# Supplementary material for: A Late Pleistocene archaic human tooth from Gua Dagang (Trader’s Cave), Niah national park, Sarawak (Malaysia)
Source: PLoS One. 2025 Dec 10;20(12):e0338786. doi: 10.1371/journal.pone.0338786 (PMC12694886; doi:10.1371/journal.pone.0338786)
Supplement: S9 Fig — (DOCX) [file pone.0338786.s009.docx]

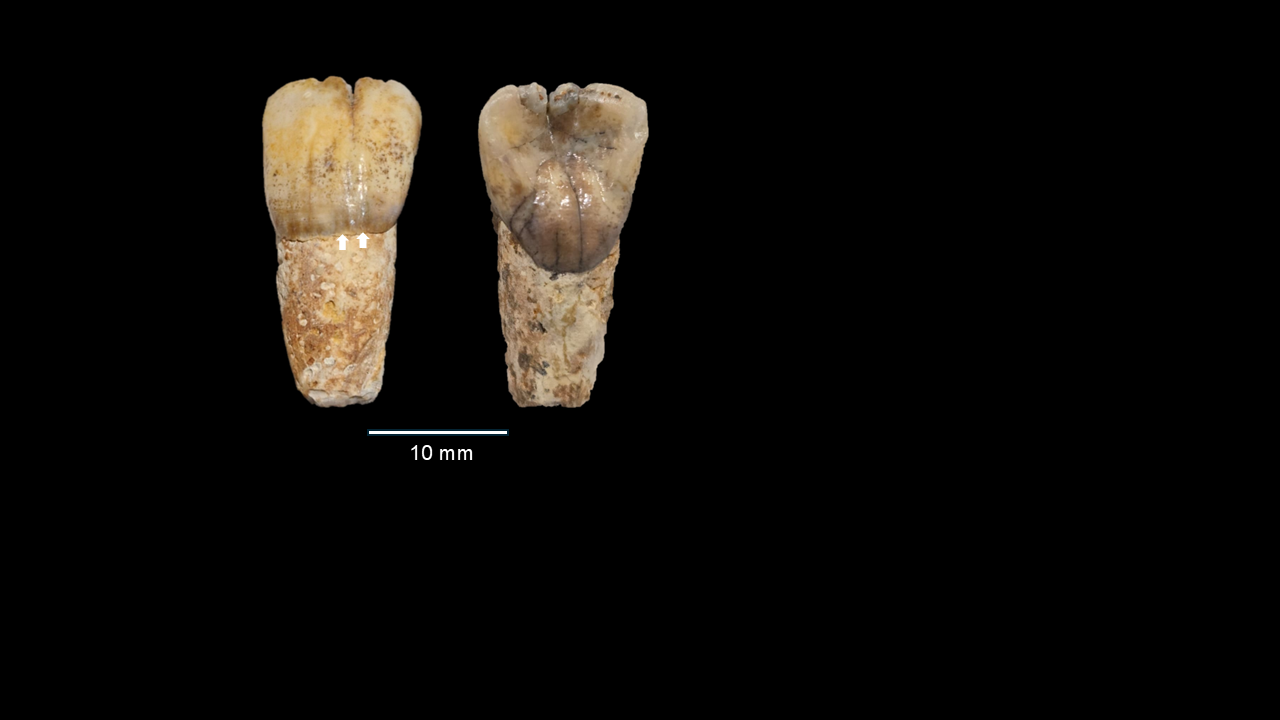


S9 Fig. **SMD-TC-AA210 higlighting the location and extent of the labiogingival notch (white arrows).** The tooth was photographed in 2025 after the removal of 3 mm from the root apex for ancient DNA testing.
